# Application of MOF-based nanotherapeutics in light-mediated cancer diagnosis and therapy

**DOI:** 10.1186/s12951-022-01631-2

**Published:** 2022-09-24

**Authors:** Dan Zhao, Wang Zhang, Shuang Yu, Si-Lei Xia, Ya-Nan Liu, Guan-Jun Yang

**Affiliations:** 1 School of Marine Science, Ningbo University, Ningbo, 315211 Zhejiang China; 2 Department of Food Science and Engineering, Ningbo University, Ningbo, 315211 China

**Keywords:** Light-mediated, Metal–organic frameworks, Nanotherapeutics, Cancer diagnosis, Therapy

## Abstract

Light-mediated nanotherapeutics have recently emerged as promising strategies to precisely control the activation of therapeutic reagents and imaging probe both in vitro and in vivo, largely ascribed to their unique properties, including minimally invasive capabilities and high spatiotemporal resolution. Nanoscale metal–organic frameworks (NMOFs), a new family of hybrid materials consisting of metal attachment sites and bridging ligands, have been explored as a new platform for enhanced cancer diagnosis and therapy due to their tunable size, modifiable surface, good biocompatibility, high agent loading and, most significantly, their ability to be preferentially deposited in tumors through enhanced permeability and retention (EPR). Especially the light-driven NMOF-based therapeutic platform, which not only allow for increased laser penetration depth and enhanced targeting, but also enable imaging-guided or combined treatments. This review provides up-to-date developments of NMOF-based therapeutic platforms for cancer treatment with emphasis on light-triggered therapeutic strategies and introduces their advances in cancer diagnosis and therapy in recent years.

**Graphic Abstract:**

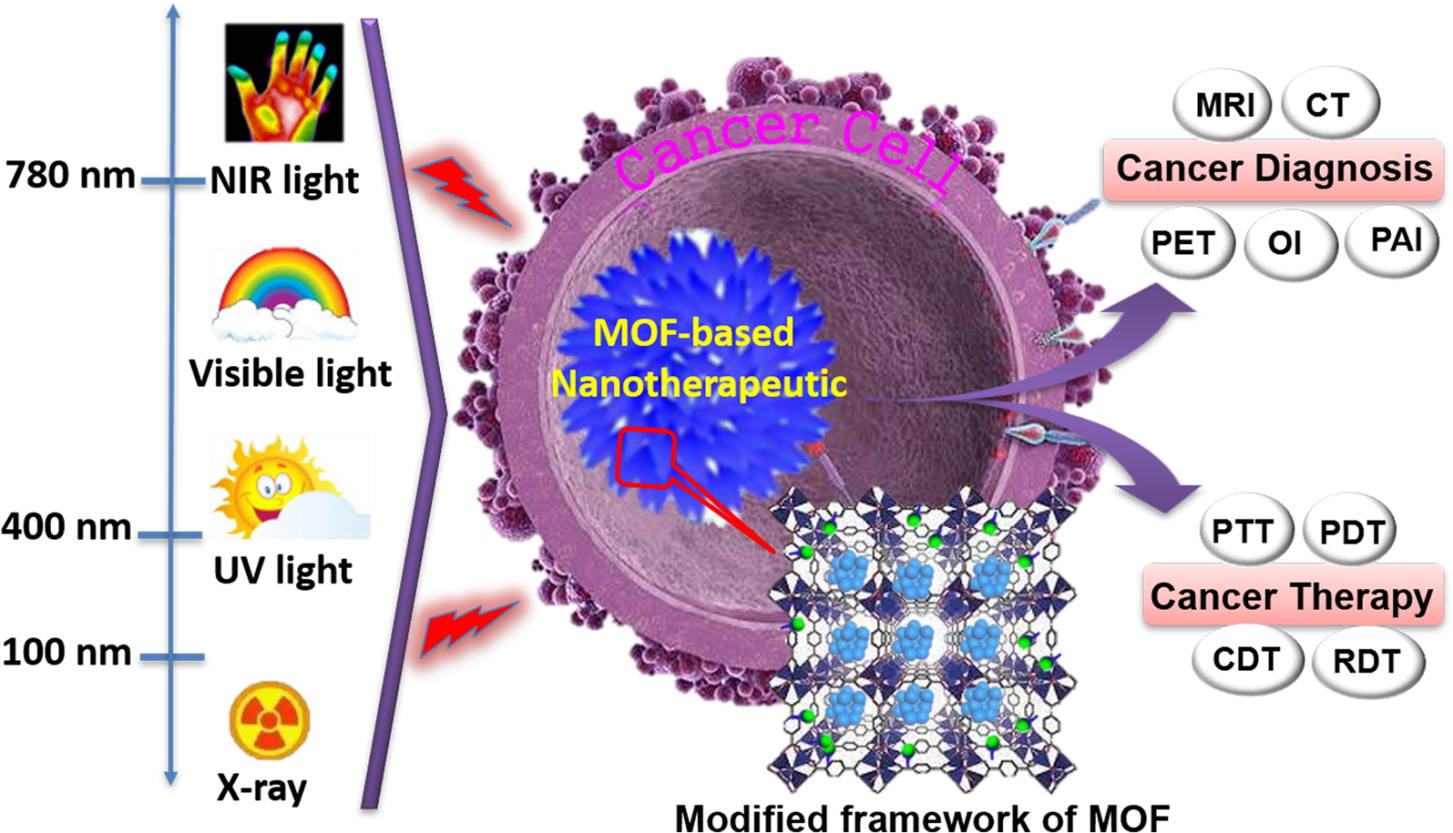

## Background

Cancer is one of the main threats to human health. Traditional tumor treatments are not ideal due to their toxicity and side effects, which will seriously affect patient compliance and survival [1–4]. How to innovate treatment methods improve drug therapeutic efficacy and reduce side effects has always been the important topics in life science. Light-mediated treatment has attracted increasing research interest in the field of cancer treatment due to its advantages of being less invasive, well-controlled, less toxic side effects and significant therapeutic effects [5]. For example: (1) photosensitizers can be selectively concentrated at the cancerous site through passive or active targeting, effectively improving treatment efficiency; (2) the use of laser-activated therapy can effectively carry out specific treatment, targeting only the lesion site while avoiding causing damage to normal tissues; and (3) non-invasive and low impact on the body. To date, therapeutic strategies employing light as a remote-control tool have received considerable attention for their specific selectivity and spatiotemporal precision.

The fusion of light functions with nanomaterials to fabricate light-mediated nanotherapeutic agents for cancer diagnosis and therapy has shown a great prospect in precision medicine, such as quantum dots, mesoporous silica, metal oxides, upconversion materials and so on [6–12]. However, these nanomaterials are usually non-biodegradable and have the potential for long-term toxicity. Therefore, the design of nanomaterial-based novel nanomedicines with high phototherapeutic efficiency still remains challenging [13–15]. Nanoscale metal–organic frameworks (NMOFs) constructed of metal-containing inorganic units and organic multi-complex linkers through coordination interactions [16–18]. Because of their excellent drug loading capacity, adjustability of the component units and good biocompatibility, NMOFs show great potential in the field of biomedicine and have been widely utilized. The tunability of NMOF components (e.g., ligands and metal nodes) facilitates the introduction of therapeutic modules for tumor treatment; the high porosity of NMOF provides a nanoplatform for the storage and delivery of various drugs, thus allowing the integration of multiple therapeutic modalities; and the functionalization of NMOF allows the enrichment of tumor tissues by active or passive targeting, thereby enabling tumor-targeted therapy. Based on these advantages, NMOFs-based cancer therapeutic nanodrugs are emerging as a new class of cancer treatment strategies [19–21].

**Fig. 1:**
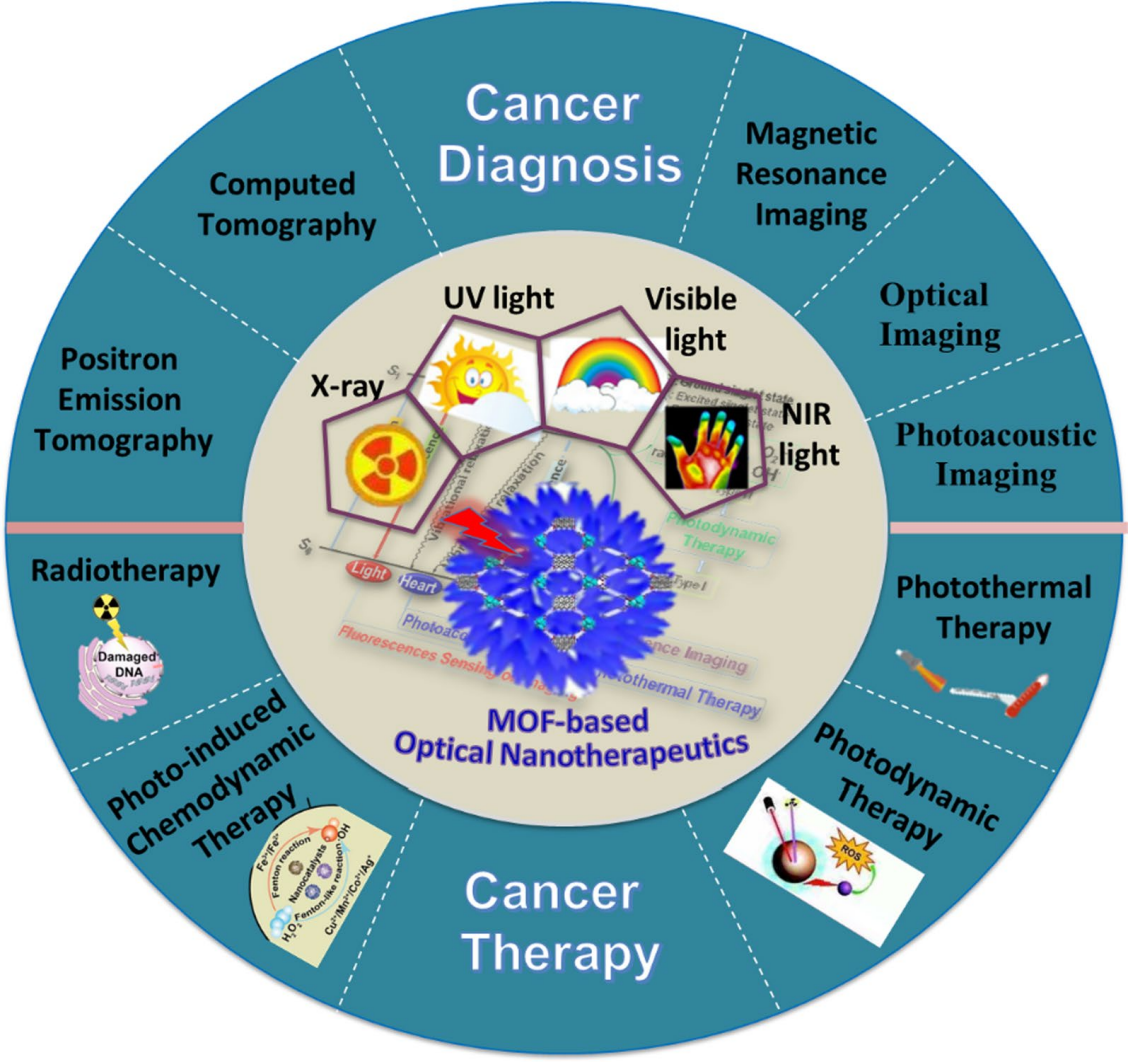
Main scope of this perspective regarding the use of MOF-based nanotherapeutics for light-mediated cancer diagnosis and therapy

Recent years witness that the enormous MOFs and their modifications leads to a concept of customized materials: that is, materials with functions tailored to specific applications, such as anticancer therapy. Therapeutic or therapeutic nanomedicine is an emerging concept where the same vehicle is both a therapeutic and an imaging (diagnostic) agent; and this is where MOFs are also seen as a promising platform [22–24]. This review summarizes the recent applications of photo-functionalized MOF nanotherapeutics in cancer diagnosis and treatment (Fig. 1). First, the general photophysical processes of light-mediated MOF-based nanotherapeutics for biomedicine-available considerations are briefly summarized. Then, NMOFs as phototherapeutic agents for cancer diagnosis and therapy are discussed. It is also briefly described that various light-mediated therapeutic mechanisms in relation to their structure-performance. Furthermore, the promise and several key issues of this field are indicated, that we hope will stimulate more interest in investigating the potential of MOF-based therapeutics for clinical applications.

## Light-mediated process of MOF-based therapeutics

Light-mediated treatment is mainly achieved by using specific wavelengths of laser light to irradiate phototherapeutic agent materials to selectively damage cancer cells and achieve tumor treatment effects [25–27]. The luminescent characteristics of MOFs make them an important sub-category of MOFs in which photon emission happens initiated by the absorption of radiative excitation energy [28].

**Fig. 2:**
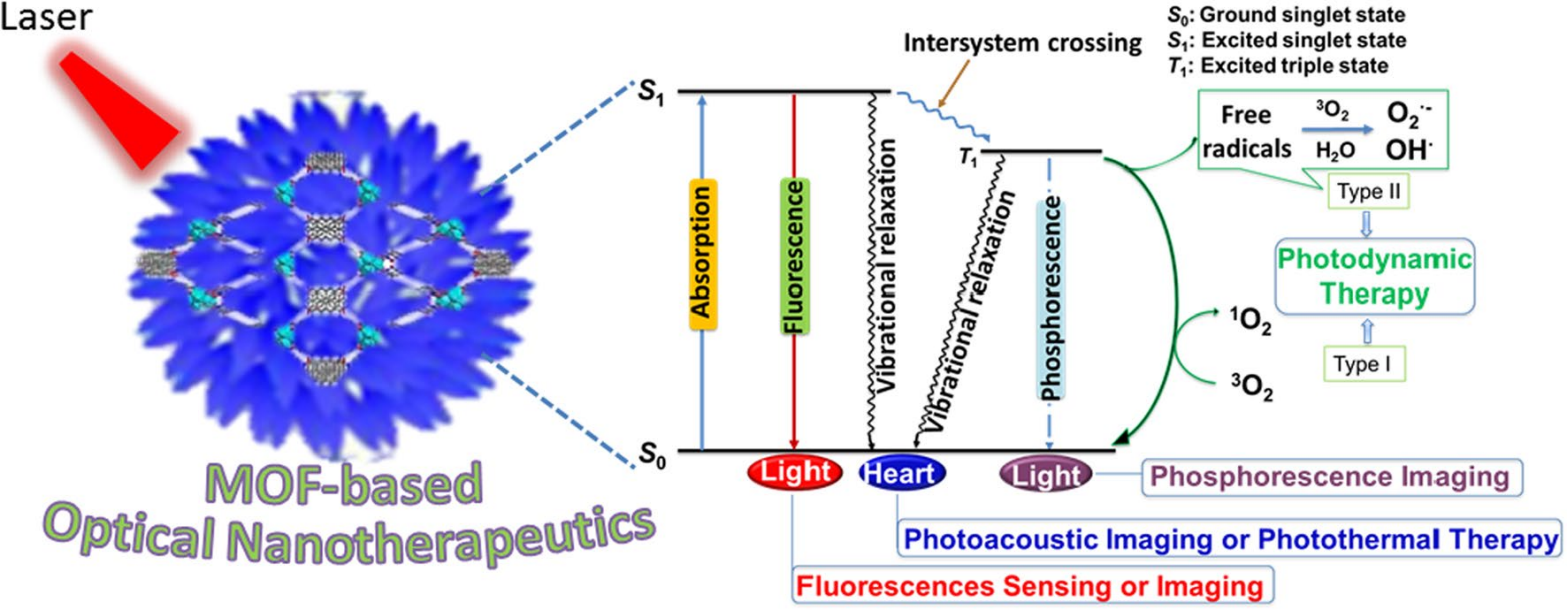
Schematic diagrams of the photophysical processes of MOF-based optical nanotherapeutics

The photophysical process of MOF-based nanotherapeutics is shown in Fig. 2. The energy in the absorption process is light and the pathway of relaxation from the excited states accompanying the emission of photons [6]. The photosensitizer (PS) absorbs photons and excites the electrons from the PS ground state (*S*_0_) to the higher energy PS excited singlet state (*S*_1_), where the excited electrons end up at the vibrational energy level of *S*_1_ and are rapidly relaxed by internal conversion to the lowest vibrational energy level of the PS excited state [24, 29, 30]. During this process, the electron returns to the ground state through three main processes: fluorescence, vibrational relaxation and inter-system scramble followed by phosphorescence, where fluorescence and phosphorescence can be used for cancer sensing and imaging. For the non-radiative transient process, the excited electrons from the PS-excited *S*_1_ to an equivalent vibrational level in the PS-excited triplet state (*T*_1_) by a change in electron spin orientation. After rapid vibrational relaxation within the *T*_1_ high energy level, the PS molecule decays to the ground state by emitting phosphorescence. In the presence of a triplet state molecule near the base, especially molecular oxygen, the triplet state of PS may relax by triggering photochemical reactions drives reactive oxygen species (ROS), which are highly toxic to cells [31]. In addition, depending on the type of reactive oxygen species (ROS) that are cytotoxic, two pathways can be distinguished: type I generates superoxide anion radicals (O_2_^·−^) and hydroxyl radicals (·OH) due to hydrogen extraction or electron transfer between the PS-excited triplet state and adjacent organic or oxygen molecules in the cellular microenvironment, while type II generates singlet state oxygen (^1^O_2_), where hydrogen peroxide, hydroxyl radicals and oxygen readily diffuse through the cell membrane, leading to further cellular damage [32–34].

## MOF-based nanotherapeutics for light-mediated cancer diagnosis

Luminescent characteristics together with size/shape selective properties of NMOFs facilitated the diagnosis of various diseases [35–37]. MOFs-based nanotherapeutics can be enriched at the tumor site for passive targeting through the high permeability and retention effects of solid tumors, or actively targeted through binding to tumor-specific receptors, thereby increasing the effective enrichment of imaging agents at the tumor site and enhancing the imaging effect [38]. NMOFs have many advantages and have proven to be outstanding contrast agents for magnetic resonance imaging (MRI), X-ray computed tomography (CT), positron emission tomography (PET), optical imaging, and photoacoustic imaging (PAI), all of which are useful techniques for clinical diagnosis due to their large number of metal attachment points or nodes (Table 1).

**Table 1:** Various MOF-based nanotherapeutics for cancer diagnosis

Diagnostic agents	Active ingredient	MOFs	Light source	Applications	Refs.
Gd(BDC)_1.5_(H_2_O)_2_	Gd^3+^	Gd(BDC)_1.5_(H_2_O)_2_	–	MRI	[43]
Gd-Ru	Gd^3+^	Gd-Ru	–	MRI	[44]
Mn-NMOF	Mn^2+^	Mn-IR825	–	MRI	[45]
Mn-TCPP	Mn^3+^	Mn-TCPP	–	MRI	[47]
MIL-88A	Fe^3+^	MIL-88A	–	MRI	[49]
MIL-100 MIL-101		MIL-100 MIL-101	–		
Fe-MIL-100@PB	Fe^3+^	Fe-MIL-100	–	MRI	[50]
NS@MOF-ZD2	Fe^3+^	MIL-101-NH_2_	–	MRI	[51]
FePt-MOFs-tLyp-1	Fe^3+^	MIL-101(Fe)	–	MRI/CT	[53]
Fe-MIL-53-NH_2_-FA-5-FAM/5-FU	Fe^3+^	Fe-MIL-53	–	MRI	[54]
Fe_3_O_4_@UiO-66	Fe_3_O_4_	UiO-66	–	MRI	[55]
Fe_3_O_4_@IRMOF-3	Fe_3_O_4_	IRMOF-3	–	MRI	[56]
Zr-UiO	Zr	Zr-UiO	X-ray	CT	[59]
Hf-UiO	Hf	Hf-UiO			
UiO-PDT	Zr	UiO-66	X-ray	CT	[60]
Mn/Hf-IR825@PDA-PEG	Mn^2+^ Hf^4+^	Mn/Hf-IR825	X-ray	MRI CT	[61]
Au@MIL-88(Fe)	Fe^3+^	MIL-88(Fe)	X-ray	MRI, CT and PAI	[63]
^89^Zr-UiO-66/Py − PGA-PEG-F3	^89^Zr	UiO-66	X-ray	PET	[68]
^64^Cu-ZIF-8@ DOX	^64^Cu	ZIF-8	X-ray	PET	[69]
Zr-NMOF	TCPP	Zr-TCPP	Visible light (530 nm)	Optical imaging	[73]
nano-Yb-PVDC-3	Yb^3+^ PVDC	Yb-PVDC-3	NIR	Optical imaging	[77]
H_2_L-MOF	H_2_L	H_2_L-MOF	NIR-I fluorescence (646 nm)	Optical imaging	[79]
MIL-53(Al)-NH_2_@RhB	RhB	MIL-53(Al)-NH_2_	Fluorescent light (375 nm)	Optical imaging	[80]
PS@MOF-199	TPAAQ	MOF-199	White light (400–700 nm)	Optical imaging	[81]
cal-TPP@(DCA5-UiO-66)	Fluorescent pyrene group	UiO-66	Fluorescent light	Optical imaging	[82]
UCNPs@MOF-DOX-AS1411	UCNPs	MIL-100 (Fe)	NIR (980 nm)	Optical imaging	[83]
ZGGO@ZIF-8-DOX	PersL NPs	ZIF-8	Fluorescent light	Optical imaging	[87]
BQ-MIL@cat-fMIL	BP Quantum dot (BQ)	MIL-101-NH_2_	Fluorescent light	Optical imaging	[88]
MCH	Polydopamine modified hyaluronic acid	MIL-100	NIR (808 nm)	PAI	[94]
(ZIF-8) derived carbon nanoparticles	–	ZIF-8	NIR (808 nm)	PAI	[95]
Cu-THQNPs	tetrahydroxyanthraquinone (THQ)	Cu-THQNPs	NIR II (1000–1350 nm)	PAI	[96]
AuNR@- ZIF-8	AuNR	ZIF-8	NIR	PAI	[97]
UCNPs@MIL-101(Fe)	Fe^3+^ UCNPs	MIL-101(Fe)	Luminescence	MRI and UCL	[100]
MIL-100(Fe)@HA@ICG	Fe^3+^ ICG	MIL-100(Fe)	Luminescence	MRI, PAI and Optical imaging	[101]
UCNPs@ZIF-8	Ag_2_S Ag_2_Se	ZIF-8	X-ray NIR (980 nm)	CT, PAI and Optical imaging	[102]

### MRI

As a powerful non-invasive imaging technique, MRI contrast agents facilitate optimal tumor assessment by shortening the longitudinal (*T*_1_) relaxation rate of water protons and/or reducing the transverse (*T*_2_) relaxation rate to enhance MRI through positive and negative contrast agents [39].

**Fig. 3:**
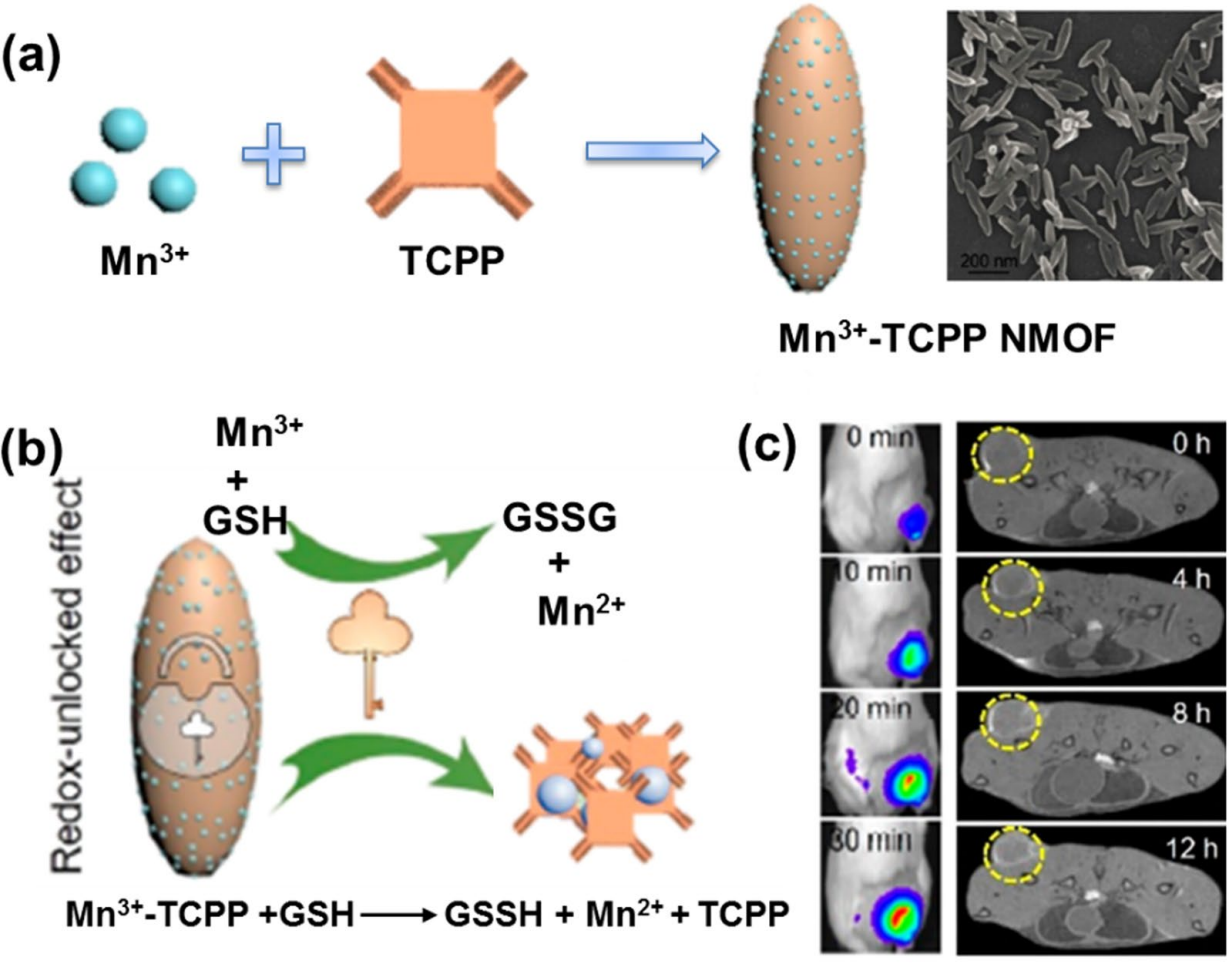
**a** Synthetic schematic and SEM photograph of Mn^3+^-TCPP NMOF; **b** mechanism of Mn^3+^-TCPP NMOF unlocking triggered by GSH; **c** in vivo fluorescence (left) and MRI (right) signals after intravenous injection with Mn^3+^-TCPP NMOF. Reproduced with permission [47]. Copyright 2019, American Chemical Society

Generally, paramagnetic metal ions (Gd^3+^ and Mn^2+^) are used to construct *T*_1_ contrast agents for MRI by chelating structures to reduce serious side effects [40]. In contrast, superparamagnetic iron oxides (SPIOs), which may lead to negative image enhancement, were chosen to construct *T*_2_-weighted MRI contrast agents. Therefore, NMOFs based on these metal ions are ideal materials for constructing MRI contrast agents [41, 42]. Early in 2006, Lin et al. reported Gd-based NMOFs as *T*_1_ contrast agents for MRI, namely Gd(BDC)_1.5_(H_2_O)_2_, with a longitudinal relaxation rate of of 35.8 s^−1^ per mm Gd^3+^, significantly higher than that of commercial *T*_1_ contrast agents [43]_._ Yin groups acquired a kind of nanomaterial that remained stable at 160 to 300 °C based on Gd^3+^ and Ru(II)[4,4′-(COOH)_2_ bipyridyl (bpy)]_3_·Cl_2_ (L_Ru_) precursors and used for MRI, namely Gd-Ru [44]. Compared with the commercial MRI contrast agent Gd-DTPA (DTPA, diethylenetriaminepentaacetic acid), Gd-Ru obtained a higher MRI contrast efficiency. Despite the fact that Gd^3+^-based NMOFs have shown good MRI capabilities, the toxicity of leached Gd^3+^ ions preclude the clinical application of these NMOFs, leading researchers to look for other metal-based MOFs for MRI. Such as, Lin and coworkers constructed Mn^2+^-based NMOF systems for MRI as the oral LD50 of Mn is 1.5 g kg^–1^ [45]. Despite the less outstanding performance of the Mn^2+^-based NMOF for *T*_1_ imaging, however, they provided an effective carrier for delivering large doses of Mn^2+^ ions result in excellent MRI both in vivo and in vitro. Liu and coworkers fabricated Mn^2+^-based MOFs with a near-infrared (NIR) dye as organic linkers, in which Mn^2+^ offers strong contrast in *T*_1_-weighted MRI [46]. The biotoxicity and biocompatibility of Mn^2+^-based MOFs were evaluated by using the standard MTT assays, which showed that Mn^2+^-based MOFs did not exhibit significant toxic effects on 293T cells, A549 cells, HeLa cells, and 4T1 cells after incubation for 24 or 48 h even with a high concentration up to 200 mg/mL. Recently, Zhang et al. reported a tumor microenvironment-responsive NMOFs system based on Mn^3+^ and tetrakis (4-carboxyphenyl) porphyrin (TCPP) (Fig. 3a) [47]. After endocytosis of the particles into tumor cells, the NMOFs were broken down by intracellular glutathione into Mn^2+^ and free TCPP, resulting in Mn^2+^-based MRI and TCPP-based fluorescence (FL) imaging (Fig. 3b and c). Furthermore, the marked differences in cell viability suggest that there are differences in the tolerance and sensitivity of various cells to GSH depletion. Of particular note, MOF-treated tumor cells (CT26, 4T1 and B16) had a lower survival rate compared to normal cells (3T3), suggesting that MOF has a tumor-specific killing effect, while avoiding toxicity to normal cells.

**Fig. 4:**
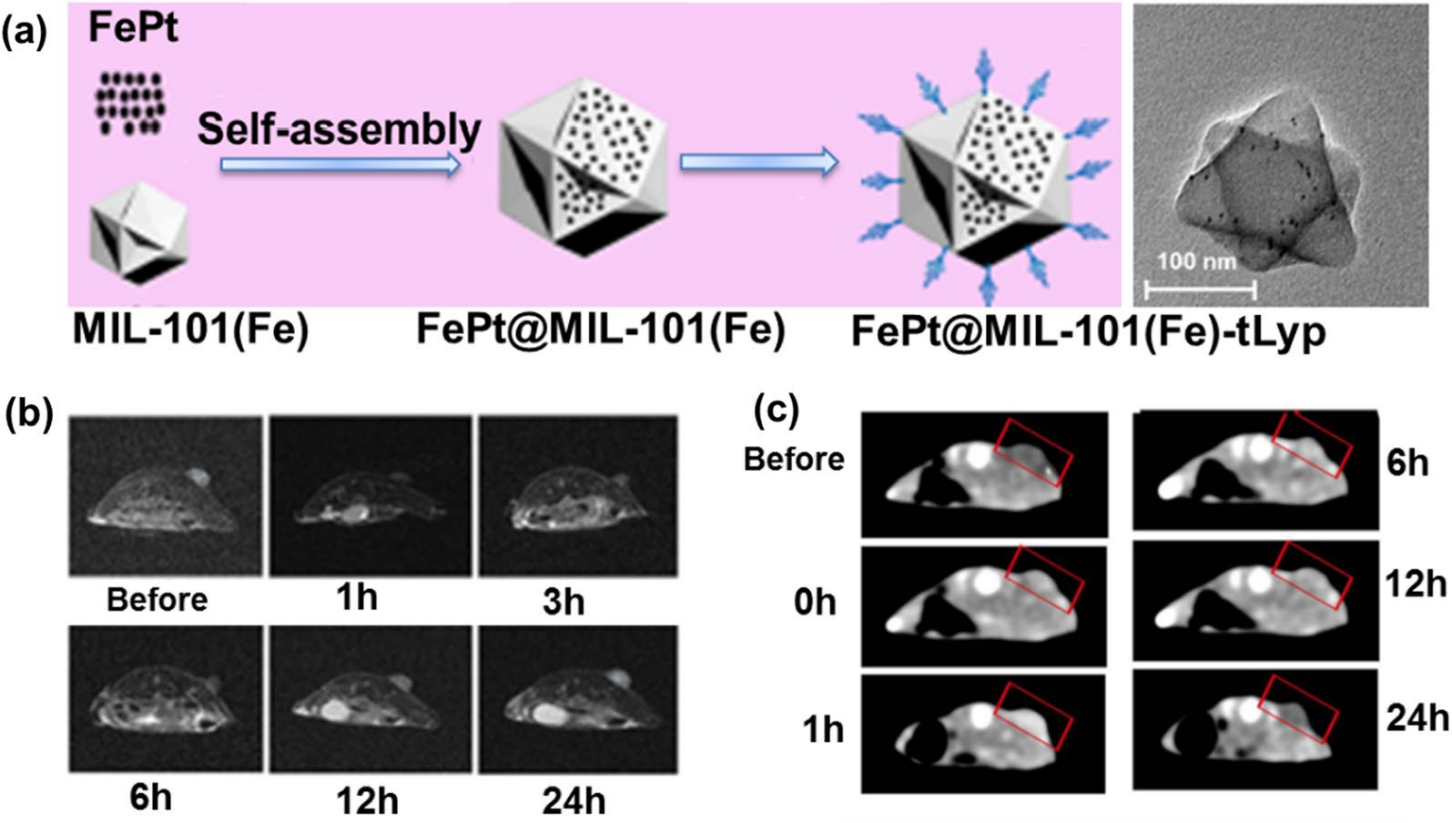
**a** Fabrication Process and TEM photograph of FePt-MOFs-tLyp-1; **b** in vivo *T*_2_-weighted MR imaging (axial plane) of a 4T1 tumor-bearing mouse at different time intervals after an intravenous injection with FePt-MOF-tLyp-1; **c** in vivo CT imaging (axial plane) of a 4T1 tumor-bearing mouse at different time intervals after intravenous injection with FePt-MOF-tLyp-1. Tumor tissue was indicated with red pan. Reproduced with permission [53]. Copyright 2020, American Chemical Society

Fe-based MOFs (Fe-MOFs) contrast agents are more common than Gd-based and Mn-based MOFs for *T*_2_-weighted MRI (oral LD50 of Fe is 30 g kg^–1^) [48]. Gref et al. have designed a series of Fe-MOFs (MIL-53, MIL-88A, MIL-89, MIL-100 and MIL-101-NH_2_ for MRI and drug delivery, demonstrating excellent therapeutic results [49]. Chen groups have synthesized bilayer NMOFs (d-MOFs) with a core–shell structure using prussian blue (PB) as the core and Fe-MIL-100 as the shell. The nanoparticles can be used for *T*_1_ and *T*_2_ dual-mode for MRI due to the presence of internal PB and external MIL-100 [50]. Zeng’s group grew MIL-101-NH_2_ on the surface of Au nanopillars and further modified the targeting peptide ZD2 to obtain an Au containing NS@MOF-ZD2 core–shell structure that can target triple-negative breast cancer [51]. Cell viability of MDA-MB-231 cells incubated with different concentrations of Au containing NS@MOF-ZD2 and the in vivo toxicity on the major organs (heart, liver, spleen, lung, and kidney) of mice proved their safe. This core–shell structure had better *T*_1_-weighted MRI, which could be attributed to the high spin of the five unpaired electrons of Fe^3+^ in this system and the active inner layer relaxation of Fe^3+^ after coordination with water. Zheng et al. encapsulated FePt nanoparticle into MIL-101(Fe) to obtained face-centered cubic FePt-MOFs-tLyp-1 with structural integrity at neutral pH levels (i.e., the bloodstream) for eradicate cancer cells (Fig. 4a). Cell viability test showed that the MIL-101(Fe)-tLyp-1 presented no significant cytotoxicity to tLyp-1 receptor-negative tumor cells (A549) and normal cells (BRL-3A). In addition, the as-prepared multifunctional FePt-MOF serve as a nanotheranostic agent for MRI/CT dual-modal imaging based on superparamagnetism (Fig. 4b and c) [52, 53].

In addition to Fe^3+^ ion, MRI can also be achieved by constructing composite structures of MOFs and Fe_3_O_4_. Other Fe-based MOFs, such as Fe-MIL-53 carrying chemotherapeutics or oligonucleotides, have been tested as MRI contrast agents [54]. Yang et al. constructed a core–shell structure using Fe_3_O_4_ as the core and UiO-66 as the shell, followed by the modification of columnar aromatics on the surface as regulatory valves on the surface to achieve MRI-guided chemotherapy with sustained release of drugs triggered by the tumor microenvironment [55]. The as-prepared core–shell nanomaterials showed negligible cytotoxicity to normal Human Umbilical Vein Endothelial Cells and Hela cells, with cell viability of almost 100% and 85% even at a high concentration of 100 µg mL^−1^. In another study, Sahu et al. demonstrated the development of a new magnetic porous system consisting of Fe_3_O_4_ core covered by a shell of IRMOF-3 with nontoxic towards HeLa cells and murine fibroblast (NIH3T3) cells for MRI [56]. The nano-agent was further modified by conjugation of a cancer targeting ligand (FA) and a fluorescent molecule (RhB). Following loading with the anticancer chemotherapeutic agent paclitaxel, NMOFs showed good therapeutic effects in HeLa cells through hydrophobic effects, high internalization through receptor-mediated endocytosis and strong *T*_2_-weighted MRI contrast due to high amounts of MRI-active Fe_3_O_4_ nanoparticles.

### X-ray CT imaging

CT, a type of tomographic imaging, is a 3D visualization of the internal structure of a scanned object depending on the absorption and transmission of X-ray [57]. In CT imaging, high atomic number elements (high Z elements) with strong X-ray attenuation properties are often used as contrast agents in order to increase the contrast between the target tissue and the adjacent tissue. Especially X-ray scintillation MOFs constructed from high atomic number metal cluster nodes (Z = 72 for Hf and Z = 40 for Zr), where the Hf(IV) and Zr(IV) cations act as antennas by absorbing X-ray photons and converting them to fast electrons by the photoelectric effect. The generated electrons scintillate/excite multiple anthracene-based optical emitters in the MOF through inelastic scattering, leading to efficient generation of detectable photons in the visible spectrum. Most importantly, NMOFs have the ability to preferentially deposit in tumors by enhancing permeability and retention (EPR) effect [58]. Large amounts of high Z elements can be easily incorporated into MOFs with extremely high payloads to form CT contrast agents. For example, the ability of two NMOFs as CT contrast agents, namely Zr-UiO and Hf-UiO, in which metal loading up to 37 wt% to Zr and 57 wt% to Hf, were demonstrated by Lin and co-works [59]. Xie et al. prepared a UiO-PDT nanosystem containing iodine-boron-dipyrromethene (BODIPY) dyes by ligand exchange [60]. The low toxicity and good biocompatibility of UiO-PDT allow it to be administered in animals without significant side effects, even at a dose of 100 mg/kg. Moreover, UiO-PDT could be enriched at the tumor site and produced optimal CT imaging after approximately 24 h of intravenous injection. Liu groups prepared core–shell NMOFs by combining IR825 with Mn^2+^ and Hf^4+^ to form multimodal imaging agents [61]. In the NMOF, each substance has its own function. Mn^2+^ ion, high-Z element Hf^4+^ ion, and the ligand IR825 are used as MRI contrast agents, enhance CT signal and radiation sensitivity, and as photothermal agents, respectively.

**Fig. 5:**
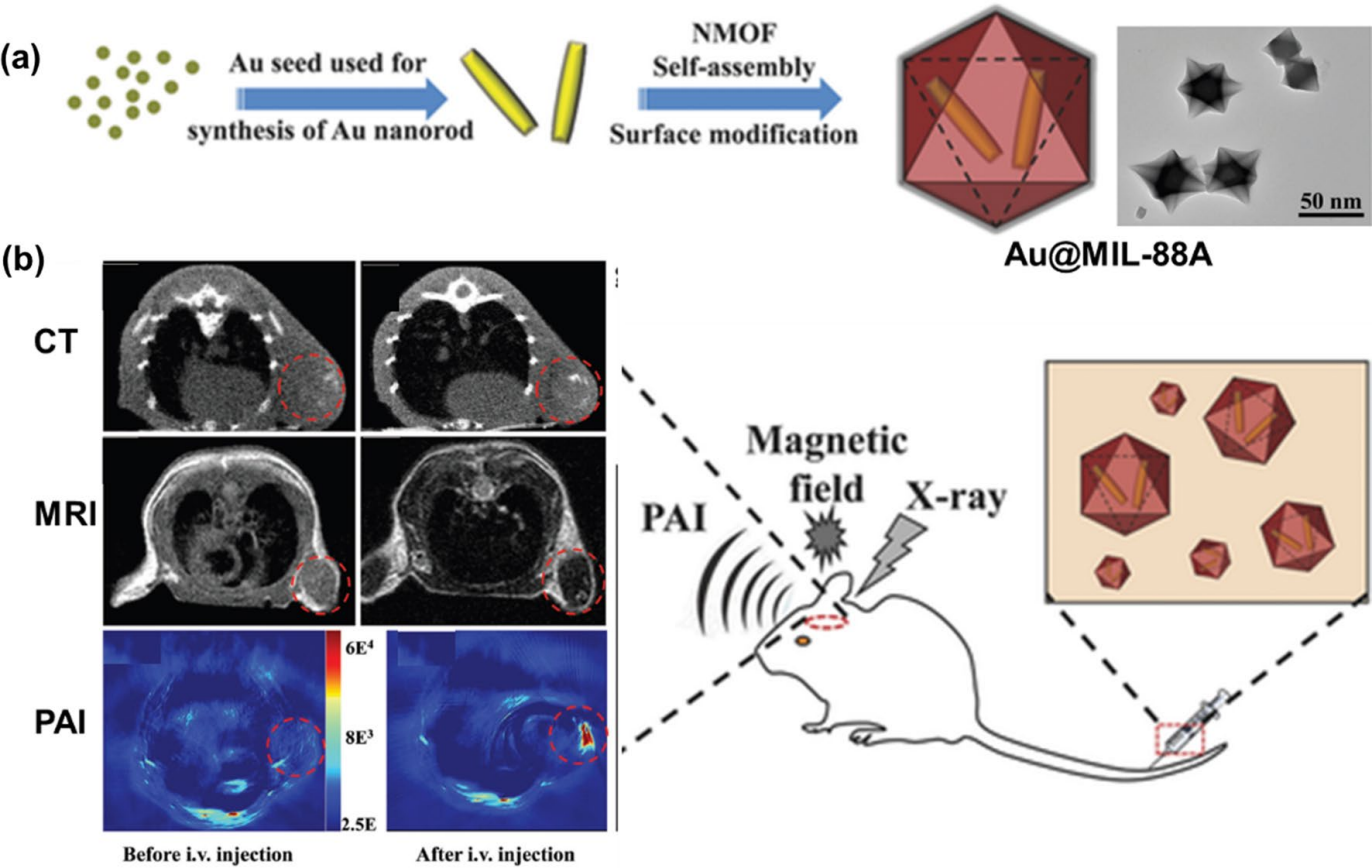
**a** Fabrication process and TEM photograph of Au@MIL-88(Fe); **b** application to multimodality imaging-based tumor diagnosis. Reproduced with permission [63]. Copyright 2016, Wiley

In addition to the addition of high Z elements to the MOFs backbone, NMOFs can be combined with other nanoparticles (e.g. gold nanorods) to obtain CT imaging capabilities [62]. Such as, the Au@MIL-88(Fe) composite system with CT, MRI and photoacoustic (PAI) tri-modal imaging capabilities was obtained by Tian group (Fig. 5a and b) [63]. Cell viability of U87MG cells was greater than 90% indicating that the as-prepared Au@MIL-88(Fe) exerted negligible cytotoxicity even at high concentrations (400 × 10^−9^ M). Moreover, the Au@MIL-88(Fe) nano-system can significantly improve the sensitivity, depth, and spatial resolution of glioma imaging and the effect of all three modalities has been demonstrated by in vitro and in vivo experiments. As can be seen from these latter examples, the addition of nanoparticles to MOF to make it intrinsically magnetically active metal is an attractive alternative, but this solution has additional components on top of MOF synthesis as well as synthesis steps, which may then complicate the large-scale manufacture of any MOF-based clinical CT contrast agent.

### X-ray PET

**Fig. 6:**
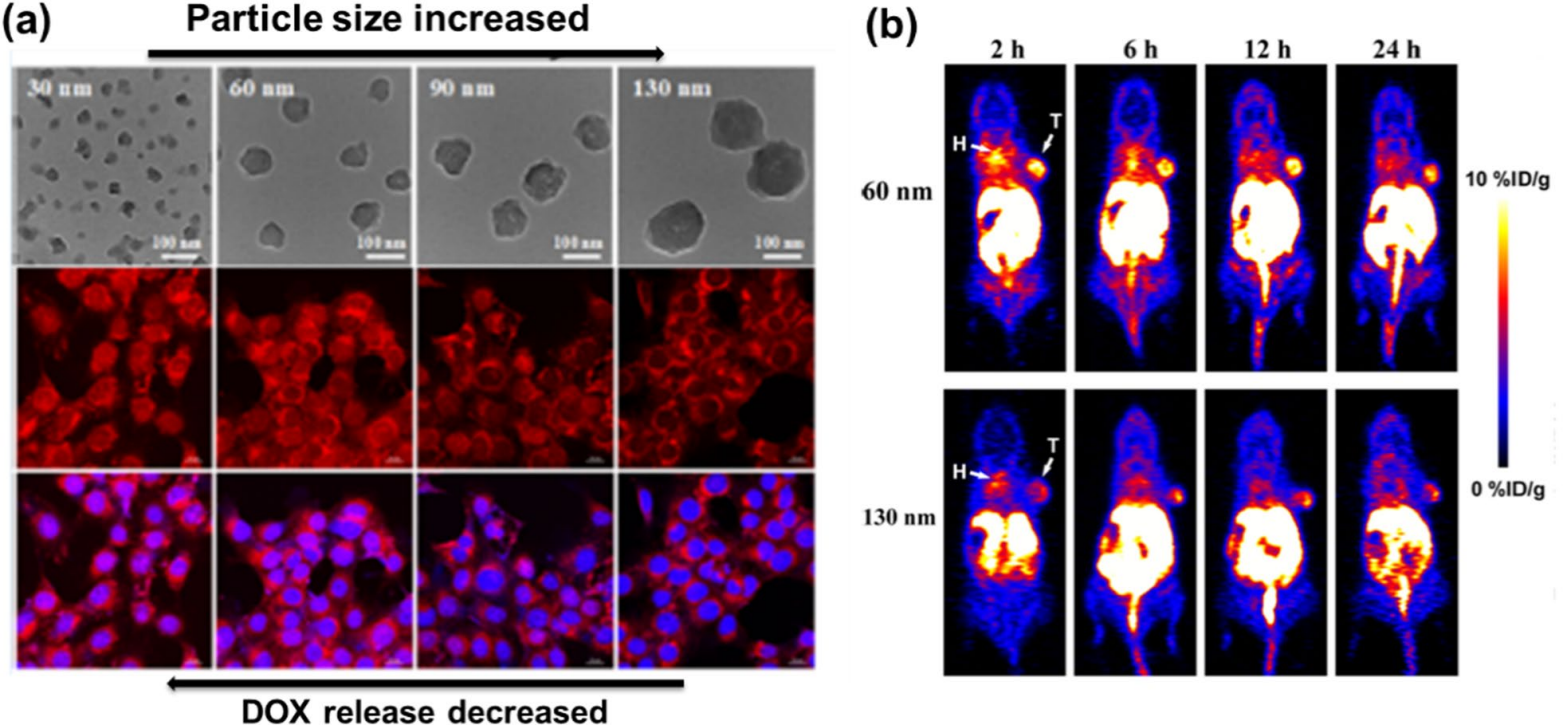
**a** Size-controlled TEM images and the DOX fluorescence intensity in nucleus decreased with the increasing size of DOX@AZIF-8; **b** DOX@AZIF-8 of different sizes exhibited significant difference in the tumor accumulation and anticancer efficacy. Reproduced with permission [69]. Copyright 2018, American Chemical Society

PET, another X-ray functional imaging method, relies on the aggregation of the substance that was labeled with a short-lived radionuclide in the metabolism for diagnostic purposes [64]. Differs from other imaging methods, PET imaging has better detection sensitivity and stronger signal penetration. NMOFs with positron imaging radioisotopes are suitable choices for this technique [65–67]. Hong groups developed intrinsically radioactive UiO-66 in which ^89^Zr as secondary building units (SBUs) was used for PET after functionalized by pyrene-derived polyethylene glycol [68]. ^89^Zr-UiO-66/Py–PGA-PEG-F3 demonstrated strong radiochemical and material stability in various biological media and PET scans traced the organ distribution of ^89^Zr-UiO-66/Py–PGA-PEG-F3 in vivo and detected an 8.2 ± 0.3% total injected dose per gram of tumor after intravenous injection for 2 h. Over 99.99% of ^89^Zr was found to remain intact after incubation of ^89^Zr-UiO-66/Py-PGA-PEG-F3 with whole mouse serum for 120 h. Based on the higher half-life of ^89^Zr (78 h) in comparison to the traditionally used ^19^F (2 h), these Zr-based PET agents have the possibility for relatively long-term use. Another example MOF-based PET was given by Liu et al., they designed a PET imaging agent by self-assembly of ^64^Cu radio-labelled and DOX loaded amorphous ZIF-8 through a rapid one-pot aqueous approach (oral LD50 = 350 μg kg^–1^ for Zn, 25 g kg^–1^ for Cu) [69]. Experimental results indicated that the rate of intracellular drug release increased with decreasing size (Fig. 6a). PET imaging showed that smaller MOF nanoparticles (60 nm) circulated in the bloodstream for a longer period of time and provided higher anti-tumor efficacy than the larger MOF (130 nm) which obtained more than 50% tumor accumulation (Fig. 6b).

### Optical imaging

Optical imaging is an imaging modality based on the detection of emitted or diffused photons after illumination with visible or near-infrared light to obtain images of cells, tissues or organs with high sensitivity and ultra-low invasiveness [70–72]. Zhang groups reported the use of Zr-based porphyrin NMOF, namely Zr-NMOF, for imaging-guided cancer therapy (oral LD50 = 4.1 g kg^–1^ for Zr) [73]. Zr-NMOF can emit fluorescent signals under 530 nm laser excitation and have demonstrated excellent imaging capabilities with high signal-to-noise ratios in both in vivo and ex vivo experiments. For optical imaging, the depth of light penetration is the main issue limiting its clinical application. Near infrared (NIR) dyes have the advantage of deep excitation light penetration and low autofluorescence and can be used to overcome the limitations of light penetration in FL imaging [74–76]. Foucault-Collet et al. reported a NIR imaging strategy based on MOFs, namely nano-Yb-PVDC-3, doped with lanthanides (Yb) and sensitizers that could be endocytosed and enable real-time NIR imaging of living cells [77]. Moreover, this work proven that MOFs materials could significantly improve the efficiency of real-time cellular imaging with rare earth elements.

**Fig. 7:**
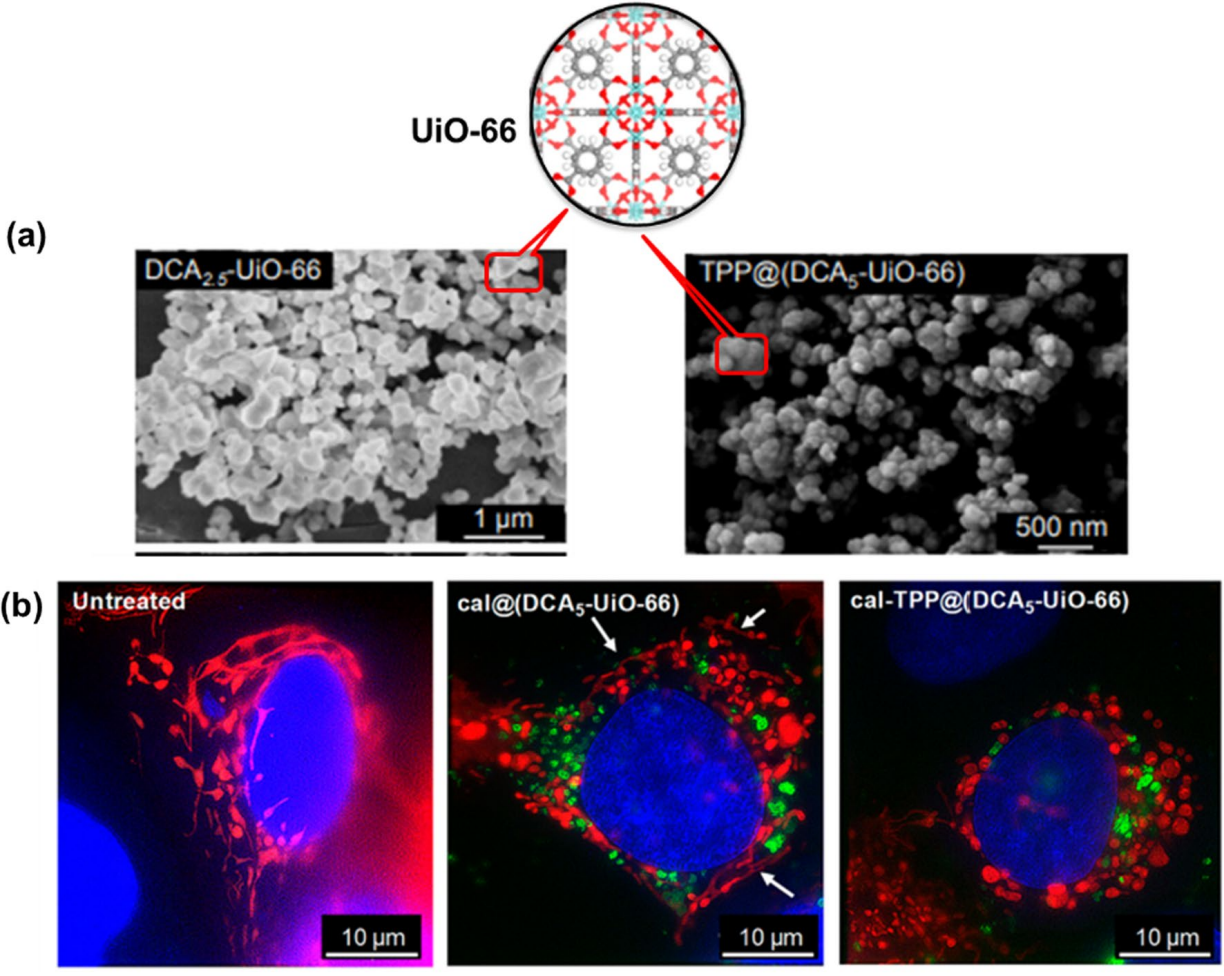
**a** Structure of UiO-66 and SEM images of synthesized DCA_2.5_-UiO-66 and TPP@(DCA_2.5_-UiO-66) samples; **b** images of untreated cells and cells treated with cal@(DCA_5_-UiO-66) and cal-TPP@(DCA_5_-UiO-66) for 8 h; mitochondria are colored in red, MOFs in green, and nuclei in blue; white arrows indicate stringy mitochondria. Reproduced with permission [82]. Copyright 2020, American Chemical Society

Associating MOFs with fluorescent dyes, PSs or fluorescent drugs is an effective way to broaden the spectral range of MOFs [78]. In addition, this strategy is also very effective in preventing aggregation-induced quenching and achieving consistent luminescence characteristics under harsh conditions through the confinement effect within the pores of the MOFs, thus improving the photoluminescence performance and efficiency of the fluorescent species [79]. As an example, Liu group demonstrated the incorporation of Rhodamine B (RhB) that serves as a fluorescent imaging agent within the framework of MIL-53(Al)-NH_2_ by a one-step approach when using RhB as aqueous solution [80]. The acquired red fluorescent NMOFs were applied for in vitro imaging of mouse gastric cancer 803 (MGC-803) cells and human airway smooth muscle cells (HASMC), and permitted in vivo imaging of thymus-free nude mice with good stability, biocompatibility and high imaging efficiency, avoiding interference from autofluorescence. In addition, the as-synthesized fluorescent NMOFs were successfully used as nanocarriers to simultaneously load the large molecule drug tetracycline hydrochloride (TCH) and the small molecule anti-cancer drug 5-fluorouracil (5-FU) with high loading efficiency and were delivered into cells. Liu et al. used MOF-199-loaded PSs wrapped around the nanoparticle surface using pozzolanium127 to obtain a therapeutically integrated nanoplatform (PS@MOF-199) [81]. The loaded PSs can perform photodynamic therapy (PDT) while giving the nanoplatform the ability to perform FL imaging. Once the nanoplatform is taken up by tumor cells, the glutathione in the tumor can disintegrate the backbone of the MOFs, releasing the PSs into contact with oxygen to perform PDT under laser irradiation, while the consumption of glutathione can further enhance the effect of PDT. Thus, this system allows for FL-guided enhanced PDT. Due to the mitochondrial membrane potential in living cells and triphenylphosphonium (TPP) is a lipophilic cation that accumulates in mito chondria. Fairen-Jimenez et al. utilized the pore characteristics of UiO-66 nanoparticles to load the anticancer drug dichloroacetate (DCA) and then surface functionalized with a TPP targeting unit (Fig. 7a) [82]. They reported that MCF-7 human breast cancer cells demonstrated changes in mitochondrial morphology when it treated with the targeted MOF system, cal-TPP@(DCA5-UiO-66), the elongated reticular network of mitochondria became short, balloon-shaped and fragmented, this is the result of DCA toxicity. In contrast, the control non-targeted cal@(DCA5-UiO-66) which showed still partially stringy and reticular morphology. For the manifestation of MOF in the cells, the green fluorescence of calcein can be determined (Fig. 7b).

**Fig. 8:**
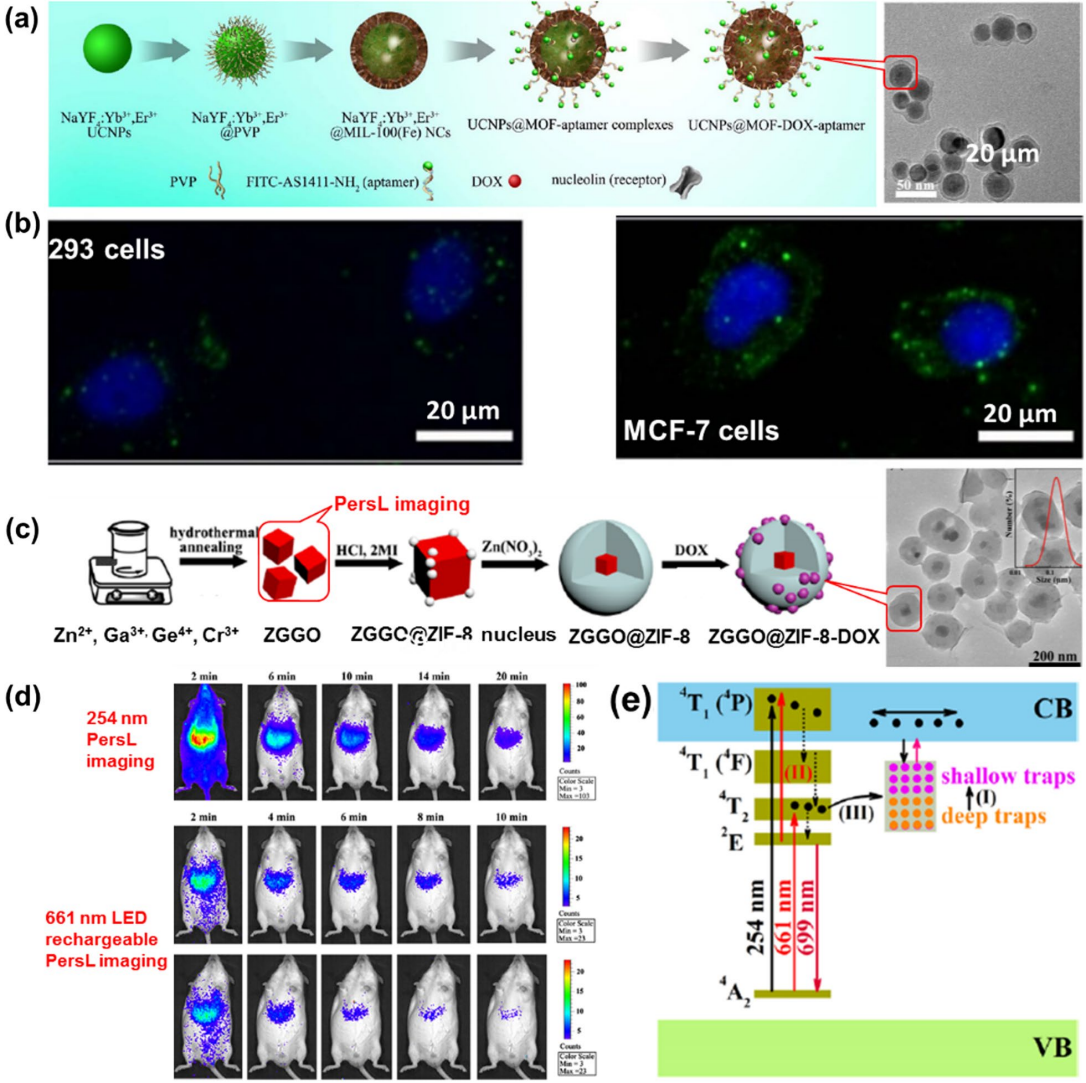
**a** Fabrication process and TEM photograph of UCNPs@MOF-DOX-AS1411; **b** images of 293 cells and MCF-7 cells incubated with UCNPs@MOF-DOX-AS1411 NCs for 1 h; **c** fabrication process and TEM photograph of ZGGO@ZIF-8-DOX; **d** in vivo NIR PersL imaging in a mouse was radiated 254 nm and 661 nm treated with ZGGO@ZIF-8 (0.2 mL, 1 mg/mL in PBS); **e** energy-level diagram for Cr^3+^-activated ZGGO. Reproduced with permission [83]. Copyright 2015, Springer Nature; [87]; Copyright 2019, American Chemical Society

Non-fluorescent reagents, including up-conversion materials and persistent luminescence (PersL) NPs, are also effectively for optical imaging by incorporating them into the MOF. By a two-step method, Deng et al. obtained core–shell nanocarriers UCNPs@MOF-DOX-AS1411 based on the mesoporous MIL-100 (Fe) shell and up-conversion luminescent NaYF_4_:Yb^3+^/Er^3+^ NPs core (Fig. 8a) [83]. The obtained nanocarriers exhibit a unique up-conversion green emission under laser excitation at 980 nm, providing UCNPs@MOF-DOX-AS1411 with the possibility of optical imaging of biological probes in vivo (Fig. 8b). The cytotoxicity of the obtained nanocarriers were evaluated by incubated with 293 cells and the standard MTT assay. At different concentration ranges, over 94% cells viabilities were observed, indicating that UCNPs@MOF NCs had no significant cytotoxic effects at all doses. Also capping upconversion NPs in MOFs, Sahu group synthesized core–shell nanoplatforms (UCNPs@ZIF-8/FA) encapsulated with FA for targeting, fluorescent imaging and pH-responsive drug release by a one-post method [84]. In this study, ZIF-8 wrapped around FA was directly encapsulated on UCNPs (NaYF4: Er^3+^, Yb^3+^) to form a monodisperse nanocomposite, in which up-conversion particles as optical imaging elements and had higher resolution images. PersL is a distinct optical phenomenon in which light can persist for hours or seconds after leaving the excitation source and the principle of optical imaging using this light is different from that of fluorescence or phosphorescence [85, 86]. Particularly, NIR PersL NPs have intrigued researchers because they can serve as optical probes bioimaging systems with preponderances of low irradiation damage, auto-fluorescence-free, and deep tissue penetration. Recently, Lv’s group obtained a multifunctional nanoplatform, ZGGO@ZIF-8-DOX, with Cr-doped zinc gallogermanate (ZGGO) NIR PersL NPs as the core and ZIF-8 as the shell with dual functionalities for auto-fluorescence-free NIR PersL imaging and the pH-responsive drug delivery, thus it has great potential in tumor theranostics (Fig. 8c) [87]. The cytotoxicity of ZGGO@ZIF-8 NPs were evaluated by incubated with murine breast cancer (4T1) cells and the standard MTT assay. After 24 h incubation with ZGGO@ZIF-8, the cell viability maintained over 85% indicated the ZGGO@ZIF-8 NPs possessed low toxicity and good biocompatibility in cells (Fig. 8d). The imaging mechanism in ZGGO NP was attributed to the ^2^E → ^4^A_2_ transition of Cr^3+^ (Fig. 8e). Compared to other imaging methods, this platform offers higher resolution and deeper tissue penetration. However, the integration of PersL NPs and MOF was currently in infancy.

In addition to the above methods, NMOFs can also be used to covalently link fluorescent molecules with functional groups of ligands for FL imaging purposes [88]. NMOFs were then covalently linked to polyethylene glycol (PEG) and folic acid (FA) by covalently linking the amino functional groups on the surface of the MOFs with carboxyl functional groups to increase the active targeting ability of the nanoparticles. The amino groups on the surface of the MOFs are then covalently linked to the carboxy-functional capped PEG and FA to increase the active targeting ability of the nanoparticles. The nanoplatform (BQ-MIL@cat-fMIL) can be used not only to monitor the enrichment of material at the tumor site by in vivo FL imaging prior to treatment, but also to reflect the change in fluorescence intensity of the tumor tissue after treatment. The successful construction of BQ-MIL@cat-fMIL also demonstrates the unique advantages of NMOFs in building a multifunctional treatment platform.

In summary, these examples of luminescent MOFs and composites highlight the wide range of strategies that can be used to prepare bioimaging materials. The use of intrinsically luminescent linkers, such as porphyrins, is highly attractive due to their simplicity, but many organic fluorophores do not possess the highly symmetrical structures that are often required for the synthesis of ordered porous MOFs. Similarly, the coordination chemistry of lanthanides, subject to steric factors, often leads to denser structures with limited porosity. Therefore, for imaging and drug delivery binding, post-synthesis binding to fluorophores or hybridization is often the more favorable approach.

### Photoacoustic imaging (PAI)

PAI is a new non-invasive and non-ionizing biomedical imaging method that has been developed in recent years. When a pulsed laser is irradiated into a biological tissue, an ultrasound signal will be generated in the light absorption domain of the tissue, and this ultrasound signal generated by light excitation is called a photoacoustic signal [89–91]. PAI combines the advantages of high selectivity in pure optical tissue imaging and deep penetration in pure ultrasound tissue imaging, resulting in high resolution and high contrast tissue images, and in principle bypassing the effects of light scattering and breaking through the “soft limit” of high-resolution optical imaging [92, 93].

**Fig. 9:**
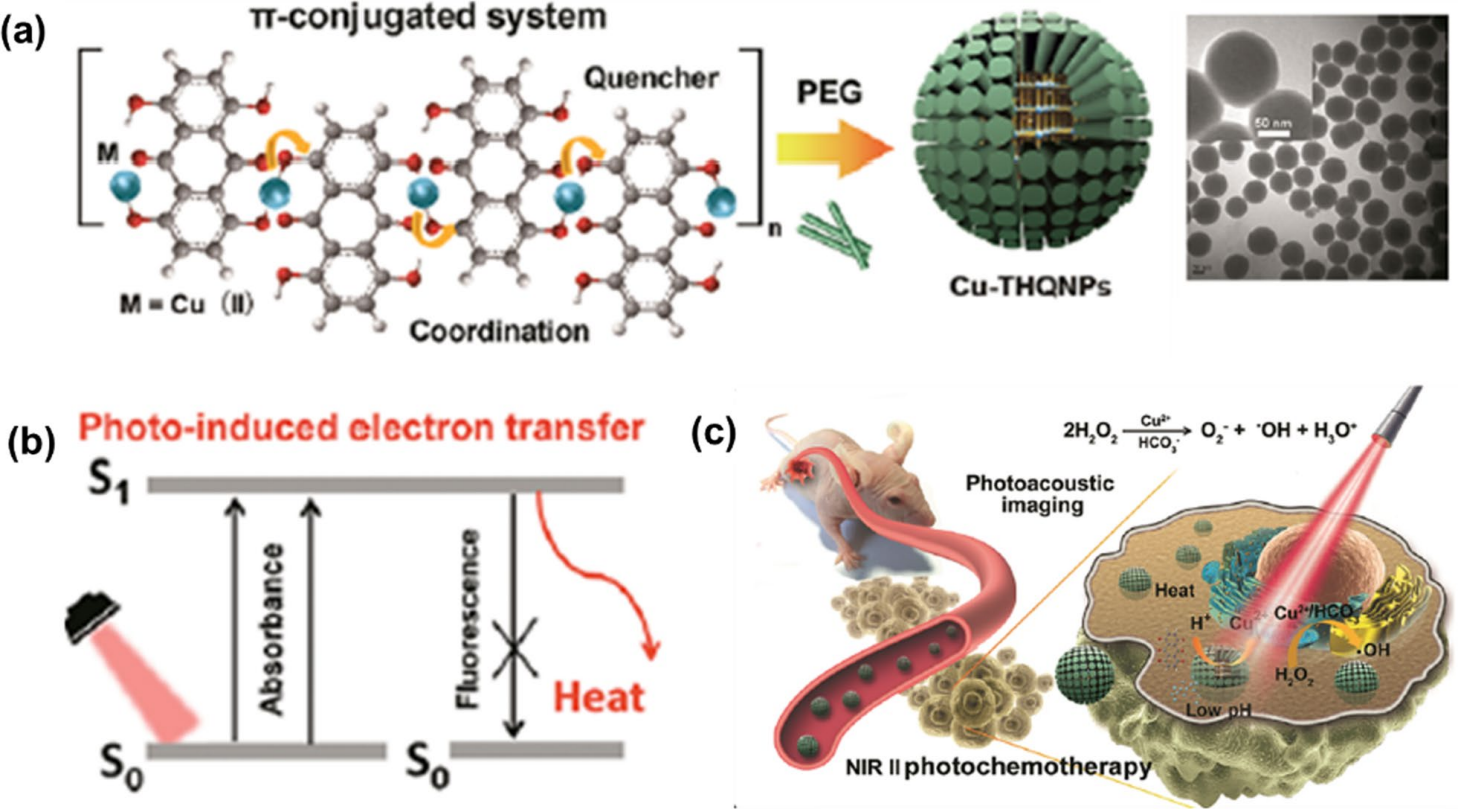
**a** Synthesis procedure and TEM image of Cu-THQNPs; **b** photoenergy to heat conversion mechanism of Cu-THQNPs; **c** schematic diagram of the behavior of Cu-THQNPs upon the 1064 nm laser irradiation in vivo. Reproduced with permission [96]. Copyright 2018, American Chemical Society

High porosity, abundant metal sites and host molecule loading capacity allow NMOFs to be designed as efficient PAI contrast agents. As an example, Chen’s group obtained a multifunctional MOF nanoplatform for PAI-guided chemotherapy/photothermal synchronous tumor therapy based on MIL-100(Fe) [94]. PAI contrast with photothermal conversion property were achieved by coating a curcumin Fe-based MOF with polydopamine (PDA) to enhance the colloidal stability and biocompatibility. By modifying with hyaluronic acid (HA)-conjugated PDA, the resulting nanoplatform were further functionalized to specifically target CD44 overexpressing tumor cells. The experimental results showed significant accumulation of the nanoparticles at the tumor site and enabled PAI-guided combined chemotherapy/photothermal treatment with high efficacy. Yang et al. employed a one-step method to obtain ZIF-8-derived carbon nanoparticles for PAI-guided cancer phototherapy, and investigated the effect of nanoparticle size on the comparative ability of phototherapy and PAI [95]. The result nano-system can act as a photothermal and PSs agents to generate both heat and ROS. In vivo PAI monitoring has shown that the system can induce efficient tumor ablation under NIR laser irradiation. Furthermore, the increase in nanoparticle size provides more phototherapeutic effect and PAI signal intensity. Subsequently, a new metal–organic nanotherapeutic agent (Cu-THQNPs) was constructed by Yuan et al. though using organic dyes, tetrahydroxyanthraquinone (THQ), as linker coordinated with Cu^2+^ ions, that serving as dual-mode therapeutics for PAI-guided photothermal/chemotherapy in the NIR II window (1000 to 1350 nm of the optical spectrum) (Fig. 9a) [96]. After PEG modification, Cu-THQNPs achieve good biocompatibility and colloidal stability. Their strong absorption in the NIR II window and PET enable Cu-THQNPs to act as excellent photothermal agents with a high photothermal conversion efficiency (51.3%) at 1064 nm and also as excellent PAI contrast agents (Fig. 9b). Additionally, acidic cleavage of tumor-specific coordination bonds allows degradation of the Cu-THQNPs leading to the release of Cu^2+^ into the tumor region, enhancing anti-tumor activity by catalyzing H_2_O_2_ to ·OH (Fig. 9c). Another study obtained PAI contrast agents (AuNR@-ZIF-8) with high PAI capacity and biocompatibility by combining ZIF-8 with AuNRs [97]. AuNR@ZIF-8 core–shell nanocomposites were successfully constructed by a simple step-by-step synthetic method involving the attachment of polyvinylpyrrolidone (PVP) to the surface of AuNR and the subsequent growth of ZIF-8 on the AuNR. The generated AuNR@ZIF-8 nano-agent exhibit high NIR absorbance, good photothermal conversion and PAI efficiency due to the presence of the AuNR.

### Multi-modal imaging

Mono-modality imaging, such as FL imaging has high sensitivity but limited penetration depth; MRI has excellent 3D soft tissue detail imaging, but its limited planar resolution and low sensitivity are not suitable for cellular level imaging; CT imaging has high resolution, but its sensitivity to soft tissue is low [98, 99]. Thus, monomodal imaging provides a limited diagnostic basis and does not reflect the full range of pathological information, thus severely undermining the effectiveness of imaging techniques in cancer diagnosis. The shortcomings of a single imaging modality can be overcome by integrating multiple imaging modalities to provide more detailed and reliable information for cancer diagnosis, which is particularly important for the accurate diagnosis and effective treatment of cancer. Due to the versatile nature of NMOFs, combining different imaging modalities on the same NMOFs platform is relatively easy to achieve.

**Fig. 10:**
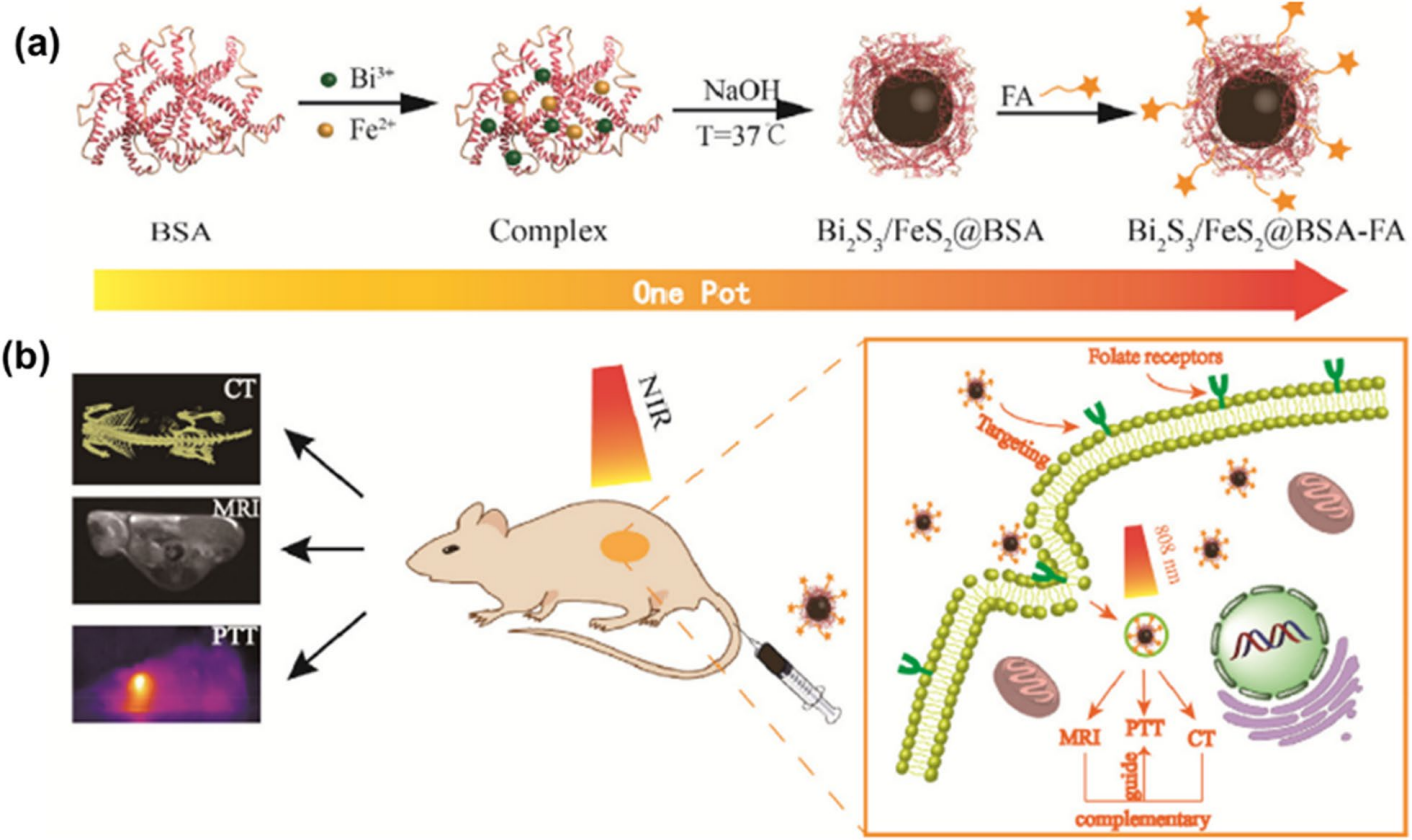
**a** The one-pot synthesis process of Bi_2_S_3_/FeS_2_@BSA-FA; **b** Schematic illustration of Bi_2_S_3_/FeS_2_@BSA-FA for MR/CT imaging and PTT. Reproduced with permission [103]. Copyright 2019, Elsevier

Tang et al. obtained core–shell nanocomposites for upconversion luminescence (UCL) and MRI dual-mode imaging by growing MOFs (MIL-101(Fe)) as a shell on surface of UCNPs [100]. Particularly, the UCNPs can be used for UCL, while MIL-101(Fe) has MRI capabilities. In vivo UCL imaging and *T*_2_-weighted MRI studies have shown that the nanomaterials can be effectively enriched in tumor sites 24 h after intravenous injection and have good UCL/MRI capabilities. Also exploiting the MRI capability of Fe-based MOFs, Chen et al. encapsulated the fluorescent molecule indocyanine green (ICG) in MIL-100(Fe) obtained MIL-100(Fe)@HA@ICG system, making the system capable of both FL imaging and photothermal ablation therapy (PAT) [101]. The MIL-100(Fe)@HA@ICG system allows for tri-modal MRI/PAI/FL imaging, overcoming the problems of penetration depth or sensitivity that exist with a single imaging modality. Kuang et al. used ZIF-8 as a framework for the simultaneous encapsulation of Ag_2_S, Ag_2_Se and UCNPs, and further integrated Au nanorods to obtain a multifunctional heterodimer based on ZIF-8 and multiple nanoparticles [102]. Under NIR excitation, Ag_2_S and Ag_2_Se can emit fluorescence at 920 nm and 1300 nm, while UCNPs can absorb light at 980 nm and emit fluorescence at 500–700 nm. High fluorescence signal expression can be observed at the tumor site even 24 h after injection of the material. At the same time, Au nanorods can be used for CT and PAI, providing high spatial resolution and high tissue penetration for tumor diagnosis. Recently, Xiong et al. designed multifunctional Bi_2_S_3_/FeS_2_@BSA-FA NHs for CT and MRI-guided tumour-targeted photothermal therapy [103]. The authors used a biomimetic mineralisation method to prepare Bi_2_S_3_/FeS_2_@BSA composites with excellent biocompatibility, which were further modified by FA to give the material active targeting capabilities (Fig. 10a). In this system, Bi_2_S_3_ and FeS_2_ provide CT and MRI functions respectively, with a high X-ray absorption coefficient of 8.02 HU-mM-1 and a transverse relative coefficient (53.9 mM^−1^ s^−1^). In vivo CT and MRI show good imaging results and the two imaging modalities complement each other to allow a fuller analysis of the tumor site (Fig. 10b).

In general, MOFs are being extensively studied as very prospective imaging agents, primarily owing to the versatility of their synthesis and the potential to integrate imaging with drug delivery. Imaging features can be inherent structural components of the MOF or guests incorporated into the pore or particle surface, either during or after synthesis, and the MOF can be readily hybridized with an array of other nanomaterials. It is this versatility that means that multiple imaging features can also be easily packed into a single particle, resulting in a multi-modal imaging agent.

## MOF-based nanotherapeutics for light-mediated cancer therapy

Tunable structure, functional capabilities, biocompatibility and biodegradability of NMOFs make them a promising platform for cancer therapy [104, 105]. For example, the specific functions of MOFs can be achieved by rational design of organic linkers, metal clusters and topologies; the performance for therapeutic application of MOFs can be tuned and improved by rational modification of the structure; the porous nature of MOFs facilitates encapsulation of therapeutic reagents to enable combination therapy. Particularly, NMOFs can be preferentially deposited in tumors by increasing permeability and EPR effects. Moreover, the incorporation of PSs into NMOFs not only tackles the binding issues of PSs, including aggregation, self-quenching and uncontrollability in vivo, but also improves loading efficiency, stability and reduces cytotoxicity. Currently, the use of light-mediated MOFs for cancer treatment is focused on photothermal therapy (PTT), photodynamic therapy (PDT), chemodynamic therapy (CDT) and so on (Table 2).

**Table 2:** Various MOF-based phototherapeutic agents for cancer therapy

Phototherapeutic agents	Photosensitizers (PSs)	Light source	Therapeutic agents	Applications	Refs.
Zr-PDI	Perylenediimide (PDI)	NIR	Zr-PDI^*−^	PTT	[111]
ZnO-CNP-TRGL	ZnO-CNP	NIR	Carbon nanoparticle	PTT	[112]
MOF@HA@ICG	Indocyanine green (ICG)	NIR	Hyaluronic acid (HA)	PTT	[94]
MIL-100@ ABTS	2,2′-azino-bis(3-ethylbenzothiazoline-6-sulfonic acid (ABTS)	NIR	·OH	PTT	[113]
Au@ZIF-8	Au	NIR	DOX	NIR-II PTT and IR/PAI imaging	[114]
CuS@Fe-MOF	CuS	NIR	DOX	PTT	[115]
Hf_6_(*μ*_3_-O)_4_(*μ*_3_-OH)_4_(DBP)_6_	Porphyrin DBP	Visible light	^1^O_2_	PDT	[119]
Hf_6_(*μ*_3_-O)_4_(*μ*_3_-OH)_4_(DBP)_6_	Porphyrin DBC	Visible light	^1^O_2_	PDT	[120]
FA@PCN-224	FA	Visible light	^1^O_2_	PDT	[121]
UCNP@PCN-224	UCNPs	NIR	Tirapazamine (TPZ) and ^1^O_2_	PDT and Chemotherapy	[124]
PCN-224-Pt	Porphyrin	NIR	^1^O_2_	PDT	[125]
BODIPY@ UiO-66	BODIPY	NIR	^1^O_2_	PDT	[126]
Zn-TCPP-BPDTE	Porphyrin and dithienylethene (DTE) derivative	UV	^1^O_2_	PDT	[127]
UCNPs/MB@ZIF-8@catalase	UCNPs and Methylene blue	NIR	^1^O_2_	PDT	[128]
oxABTS@MIL-100		Visible light	·OH	CDT	[113]
Cu-TBP	Tetracarboxyphenylporphyrin (H_4_TBP)	Visible light	·OH and ^1^O_2_	PDT and CDT	[135]
O_2_-Cu/ZIF-8@Ce6/ZIF-8@F127,	Ce6	Visible light	·OH and ^1^O_2_	PDT and CDT	[136]
^99m^Tc-Hf-TCPP-PEG	Hf^4+^ and ^99m^Tc	X-ray	ROS	RDT	[139]
Hf_6_-DBA Hf_12_-DBA	Hf^4+^	X-ray	ROS	RDT	[140]
Zr-MOF-QU	Zr^4+^ 1,4-benzenedicarboxylic acid	X-ray	ROS	RDT	[142]
d-Arg-MIL-100 (Fe)	Fe^3+^	X-ray	·OH and ^1^O_2_	RDT and CDT	[143]

### Photothermal therapy (PTT)

PTT is a treatment that uses a material which absorbs light with high photothermal conversion efficiency, injects the material into the body and irradiates it externally with light, and the material converts the light energy into heat to kill cancer cells [106]. The main mechanisms involved in PTT at the research stage are: energy transfer to the surrounding lattice in the form of atomic collisions: i.e. radiation-free relaxation processes; local surface plasma excitonic resonance effects caused by free carriers; and non-radiative compounding of excited electrons with holes through deep defects: i.e. phonon intense lattice vibrations [107–110]. In the PTT-mediated procedure, the photothermal agent is usually injected into the patient, e.g. by intravenous injection, and aggregated into the tumor by means of a highly permeable long retention effect (EPR) or a bound targeting factor. There are three main types of MOF-based nanotherapeutic agents that use the PTT principle to treat cancer: (1) MOF nanotherapeutic agents; (2) MOF-derived nanotherapeutic agents; (3) MOF-coated photothermal agent nanocomposites.

#### MOF therapeutic agents

**Fig. 11:**
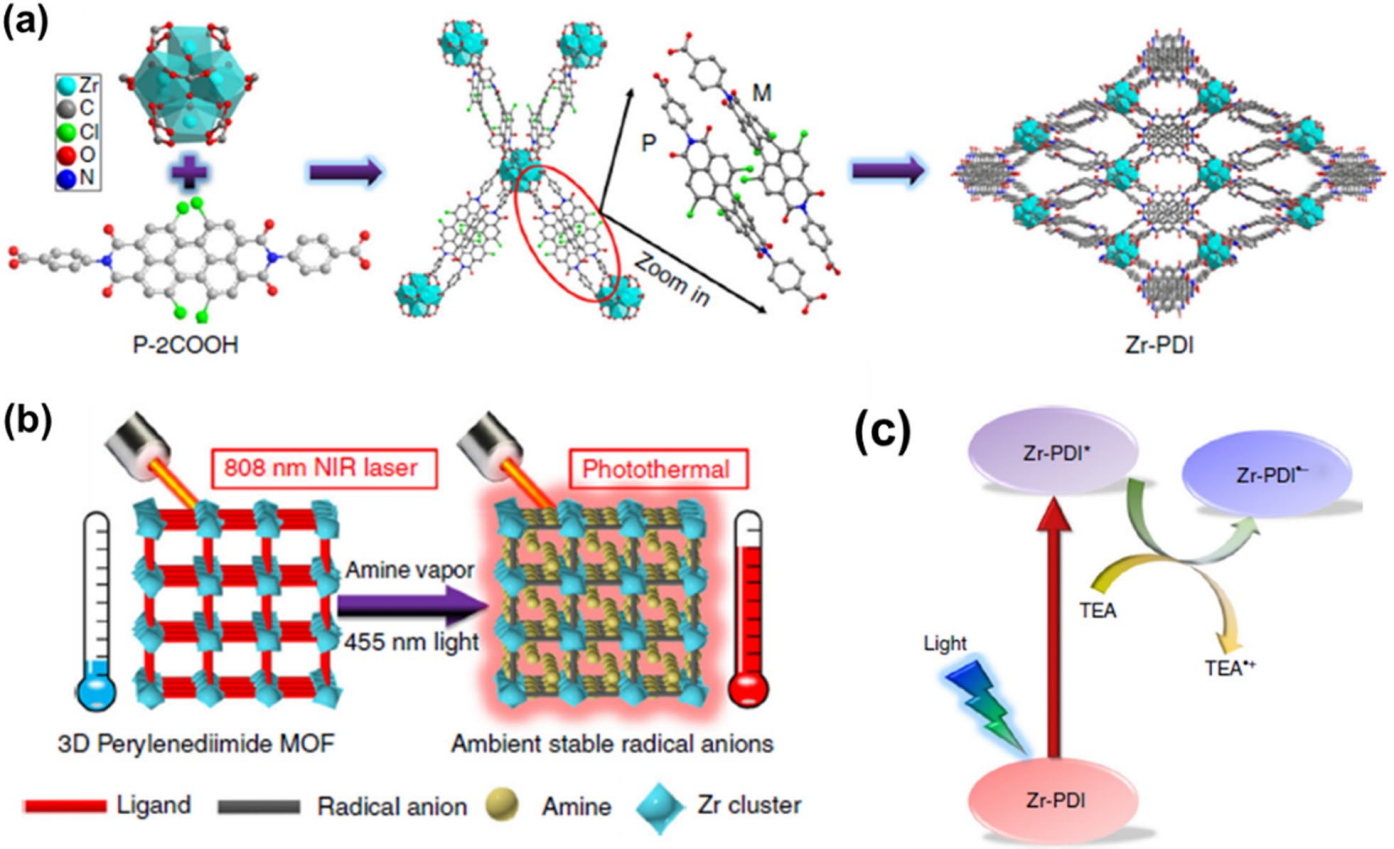
**a** Synthesis procedure and structure of 3D porous Zr-PDI; **b** illustration of photothermal conversion performance of Zr-PDI^*−^; **c** schematic diagram of the PET process between TEA and Zr-PDI, excited state Zr-PDI^*^ is reductively quenched by TEA to afford Zr-PDI^*−^. Reproduced with permission [111]. Copyright 2018, Springer Nature

MOF nanotherapeutic agents which rely on their own photothermal conversion to achieve PTT. Such as, by self-assembly of NIR dye IR825 and Mn^2+^, a MOF nanoplatform with strong NIR absorbance and high MRI contrast performance was obtained by Liu group [46]. After modification with PDA and PEG, this nanoplatform not only exhibits excellent PTT efficiency with good NIR photostability and high MRI contrast performance compared to a single IR825 molecule, but also shows effective photothermal tumor ablation with minimal long-term toxicity. Subsequently, Yin’s group reported a first 3D perylenediimide (PDIs)-based MOF, namely Zr-PDI, composed of a *N*,*N*′-di-(4-benzoic acid)-1,2,6,7-tetrachloroperylene-3,4,9,10-tetracarboxylic acid diimide (P-2COOH) ligand and Zr_6_(μ_3_-O)_4_(μ_3_-OH)_4_ clusters (Fig. 11a) [111]. Zr-PDI has excellent stability and a specific surface area of 1330 m^2^/g. The high adsorption capacity of high boiling point amine vapors can generate highly stable anionic radicals Zr-PDI^*−^ through PET. Zr-PDI^*−^, with NIR absorbance, in addition to high yield and stability, shows an exceptionally high NIR photothermal conversion efficiency (η = 52.3%) and good recyclability, which can be applied in the field of photothermal therapy (Fig. 11b and c).

#### MOF-derived nanotherapeutic agents

**Fig. 12:**
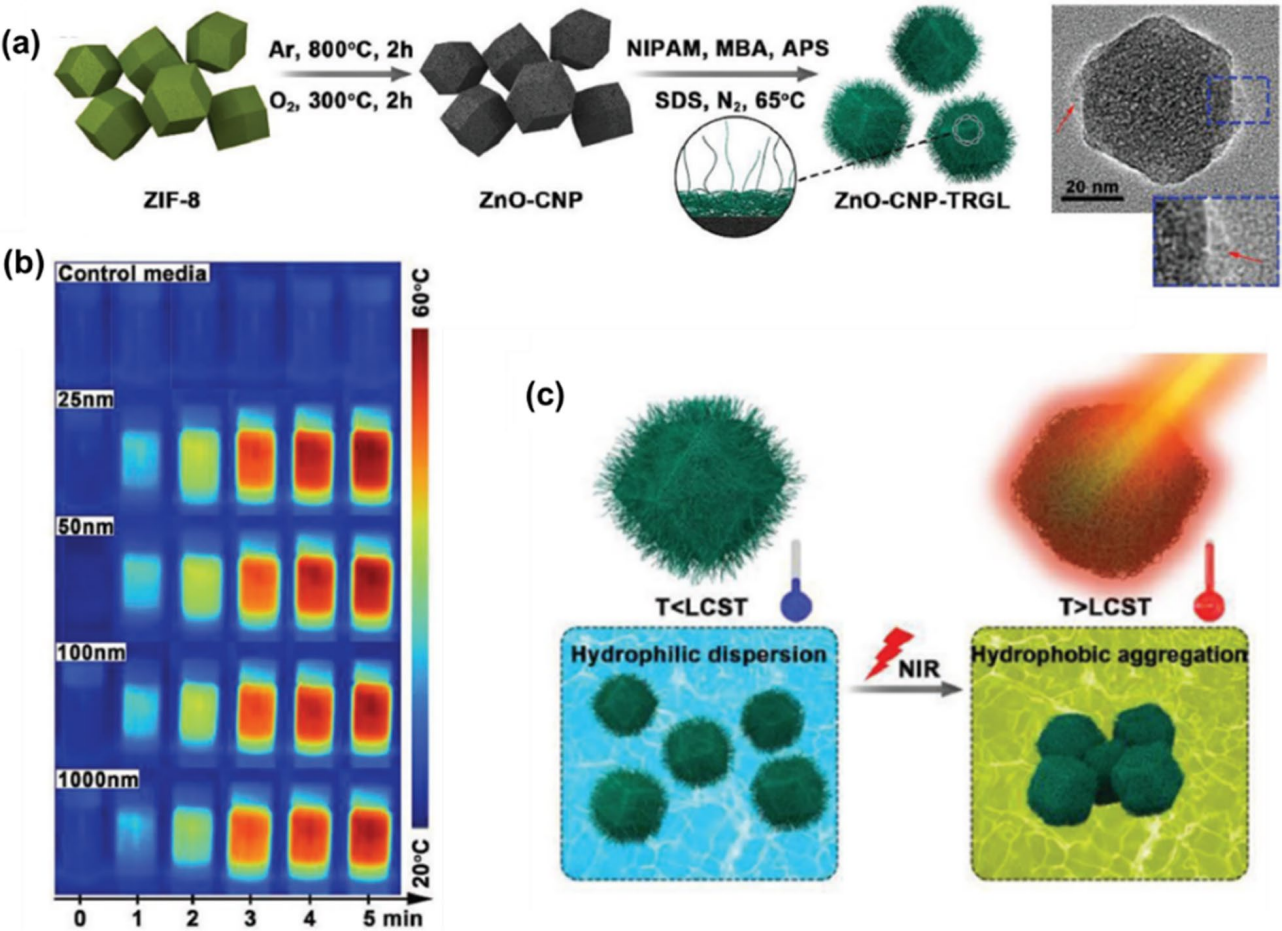
**a** Synthesis procedure and TEM image of ZnO-CNP-TRGL; **b** Photothermal images for control media and ZnO-CNP-TRGL (25, 50, 100, and 1000 nm) suspensions (50 µg mL^−1^) with different irradiation times (0–5 min) at 2 W cm^−2^; **c** Illustration images for the size transformation of ZnO-CNP-TRGL from hydrophilic dispersion and hydrophobic micrometer aggregation; LCST is lower critical solution temperature. Reproduced with permission [112]. Copyright 2019, Wiley

After different thermal and/or chemical treatments, MOFs can be used as sacrificial templates to derive various porous nanomaterials. For example, MOF-derived nanocarbons exhibit controllable porous architectures, pore volumes, and surface areas as well as high photothermal conversion efficiency. Zhao et al. reported an example of MOF-based carbon nanomaterial (ZnO-CNP-TRGL) for cancer therapy with NIR response and variable size by a subsequent annealing and sintering process under the condition of pre-synthesis of MOF, and then coated with a thermally responsive gel layer to obtain switching capability (Fig. 12a) [112]. Cytotoxicity assays showed that the cytocompatibility of MOF-based carbon nanomaterial increased with the increased particle sizes and reduced dosage when incubated with CCK-8 cells. Notably, the prepared carbon nanocomposites exhibited efficient photothermal conversion and rapid size transition from nanodispersions to micrometer aggregates under 808 nm NIR light irradiation, thus enabling the nanocomposites to generate large amounts of heat in the affected area and directly destroy proteins in tumor or bacterial cells (Fig. 12b and c).

#### MOF-coated nanocomposite photothermal agents

The therapeutic efficiency of PTT can be improved by encapsulating the photothermal agents (PTAs) in NMOFs to form a MOF-coated nanocomposite. Such as, organic dyes (ICG, IR825) with NIR absorption capability could be embedded into MOFs to formed nanocomposites to overcome poor solubility and low tumor specificity. By loading ICG into MIL-100 (Fe) and modifying with hyaluronic acid (HA), Chen’s group obtained a multifunctional nanoplatform, namely MIL-100@HA@ICG [94]. The toxicology of MOF@HA@ICG NPs was determined by incubating MCF-7 cancer cells with for 48 h. Cell survival rates of over 80% indicated that NPs had low cell cytotoxicity to MCF-7 cancer cells at NP concentrations that ranged from 0 to 250 μg/mL. Anti-tumor experiments indicate that MIL-100@HA@ICG can effectively inhibit the growth of MCF-7 tumors by improving NIR absorbance, photostability and tumor accumulation.

In addition to direct loading of PTAs, PTT can also be achieved by loading precursor compounds of PTAs with tumor microenvironmental responsiveness. As an example, Chen et al. obtained an AMP nano-agent by loading 2,2′-azino-bis(3-ethylbenzothiazoline-6-sulfonic acid (ABTS) into MIL-100 [113]. In vitro cytotoxicity assessment showed that AMP nano-agent exhibited negligible cytotoxicity to 4T1 cells in the absence of H_2_O_2_ and NIR laser irradiation. Then, the therapeutic properties of AMP nano-agent were tested in 4T1 tumour-bearing mice. The results show that in the tumor microenvironment, Fe^3+^ ions in AMP nano-agent can catalyze H_2_O_2_ to produce ·OH, and then ·OH can oxidate ABTS to oxidation state in the presence of PTT, enabling tumor microenvironment-responsive PTT and further improving the safety of PTT. In addition, the ·OH produced by this system can achieve a chemical kinetic killing effect on tumor tissue. Treatment experiments have shown that tumor tissue in vivo can be completely eliminated by AMP nanosystem.

**Fig. 13:**
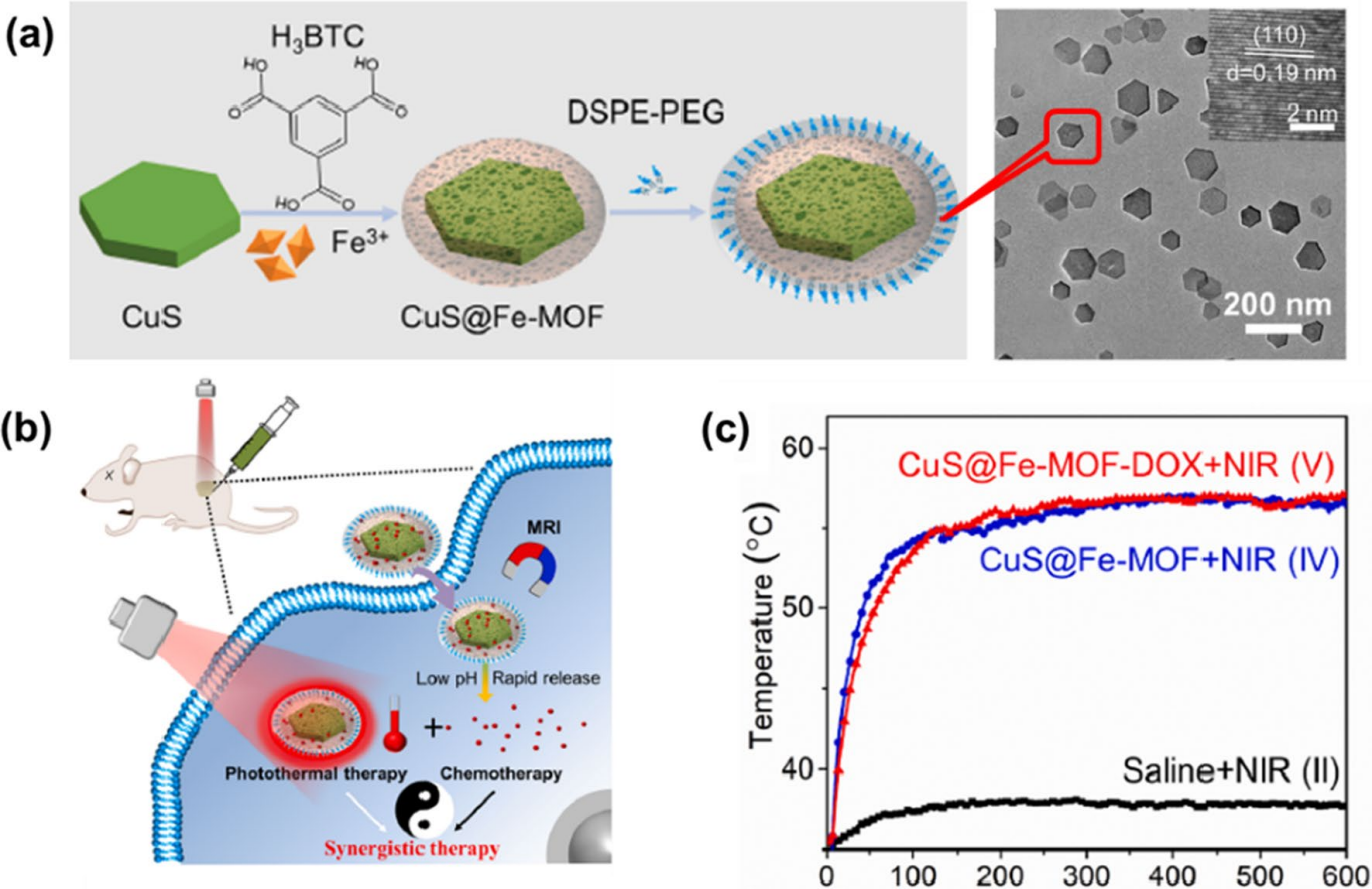
**a** Synthesis scheme and TEM image of CuS@Fe-MOF; **b** Scheme of PAT process; **c** Temperature elevation curves in the tumor site. Reproduced with permission [115]. Copyright 2019, Elsevier

Besides loading organic PTAs, another effective strategy to constructed MOF-based nanocomposites for PTT is integrating NMOFs with PTT functionality inorganic NPs. For example, by depositing ZIF-8 on Au star NPs, Lin’s group developed a yolk-shell structure of Au@ZIF-8, enabling bimodal imaging for diagnosis and chemotherapeutic treatment [114]. The group of the cells incubated with Au@MOF alone showed no significant cell apoptosis under the studied concentrations. The inherent localized surface plasmon resonance (LSPR) properties of Au have led to its use for NIR-II PTT and IR/PAI imaging. Furthermore, DOX was encapsulated in the cavity of the platform, which exhibited pH-controlled drug release because of the degradation of ZIF-8 in the acidic tumor microenvironment. Semiconductor CuS, which is inexpensive and has good photothermal effects, has attracted a lot of attention in PTT [115]. Chen et al. have obtained a new core–shell nanoplatform CuS@Fe-MOF based on a co-precipitation and assembly strategy (Fig. 13a). Higher than 85% cell viability reveals the low cytotoxicity of CuS@Fe-MOF after 24 h incubation HUVEC cells with CuS@Fe-MOF (0–0.3 g L^−1^). The integrated CuS containing nanoplatform exhibits high DOX loading capacity (27.5%), photothermal conversion efficiency (39.7%), pH-controlled drug delivery, MRI capability and good biocompatibility (Fig. 13b and c).

### Photodynamic therapy (PDT)

PDT is a non-invasive treatment that allows precise ablation of tumors. In cancer treatment, the PSs are usually enriched in the tumor tissue and the laser is then used to irradiate the tumor site to produce large amounts of ROS to kill the tumor cells [116]. The reactive species generated during the PDT process can kill cancer cells not only directly through apoptosis, necrosis or autophagy, but also indirectly kill cancer cells by destroying the tumor vascular system and causing tumor ischemia [117]. Moreover, PDT can trigger an immune response against tumor antigens, further inhibiting tumor growth and recurrence. In PDT process, there are three key factors, including light, PSs and oxygen. Local irradiation of the tumor site by laser after enrichment of PSs at the tumor site allows selective killing of tumor tissue and minimizes damage to healthy tissue. At present, a variety of MOF-based nanomaterials have been used in PDT due to their easy diffusion of ROS and the avoidance of self-quenching of PSs.

Porphyrins and their metal derivatives are commonly used as PSs in the PDT process [118]. However, these PSs still have certain drawbacks. For example, the PSs lacks targeting in vivo and its aggregation at the tumor site reduces the effectiveness of the treatment. MOFs constructed using organic ligands with porphyrin-containing structures largely overcome these problems by forming porous skeletal structures through the covalent binding of metal centers to ligands. Early in 2014, Lin et al. used a porphyrin derivative (5,15-di(p-benzoato)porphyrin, H_2_DBP) as a ligand and Hf as metal ions to obtain DBP-UiO with nanoplate morphology [119]. In this system, the DBP molecules are separated by metal nodes to avoid aggregation and quenching, and the coordination of the DPB molecules with Hf facilitates the energy transfer and hence the yield of ROS. The porous structure of the system also facilitates the diffusion of ^1^O_2_. Subsequently, the same group synthesized DBC-UiO, a more effective PDT, using 5,15-di(p-benzoato)-chlorin (H_2_DBC) as a ligand, with three times the efficiency of DBP-UiO in the production of ^1^O_2_ [120]. Zhou et al. used Zr as the metal node to synthesize a series of PCN-224 with different particle sizes based on porphyrin ligands, and investigated the effect of particle size on cellular uptake. Then, Zr6 metal cluster in the PCN-224 molecule was coordinated with the targeting molecule FA, giving it the ability to actively target FA receptors and enhancing its PDT effect [121].

**Fig. 14:**
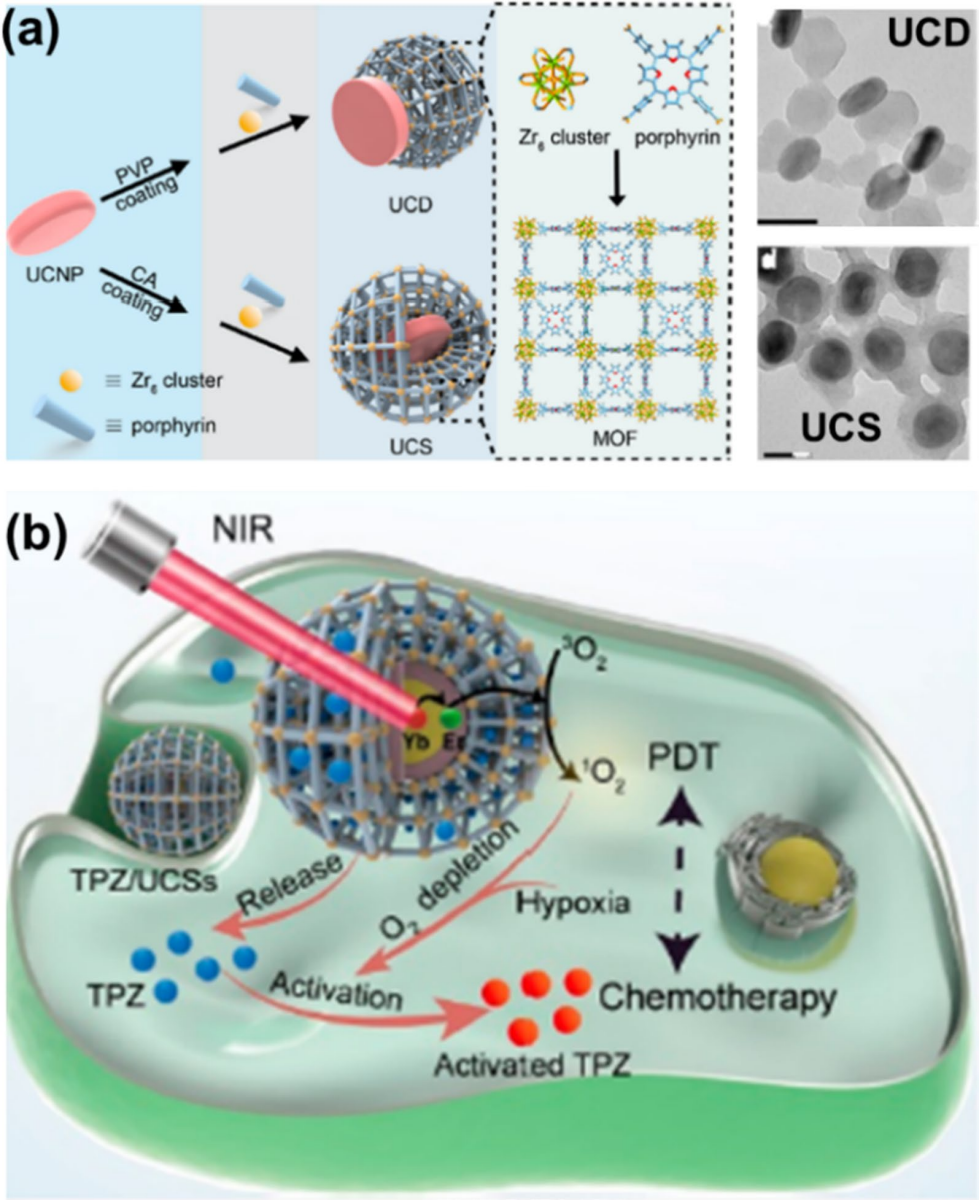
**a** Schematic illustration and TEM images of the synthesis of UCD and UCS by the surface engineering of UCNPs; **b** schematic diagram of the treatment process of TPZ/UCSs. Reproduced with permission [124]. Copyright 2020, American Chemical Society

Light, another key factor in PDT, limits the effectiveness of PDT and its ability to treat deep-seated tumors, so conventional PDT is limited to the treatment of skin tumors [122]. The NMOFs offer further possibilities to improve these constraints due to their ease of functionalization. Using the photoconversion capabilities of UCNPs, these nanocomposite systems can be excited by NIR light to produce ROS and thus achieve better PDT results. For example, Li et al. combined PCN-224 and UCNPs to obtain heterostructure and core–shell nanocomposite systems using different preparation methods, namely UCD and UCS (Fig. 14a) [123, 124]. Through conditional surface engineering of UCNPs and subsequent seed-mediated growth strategies, UCSs were synthesized in high yields. The heterogeneous structure of UCS allows for efficient energy transfer from the UCNP core to the MOF shell, which makes it possible to generate cytotoxic reactive oxygen species triggered by NIR light. By encapsulating the hypoxia-activated pro-drug tirapazamine (TPZ) in the nanopore of a heterogeneously structured MOF shell to produce the final construct TPZ/UCS, TPZ/UCSs represent a promising system to achieve improved cancer therapy both in vitro and in vivo. through a combination of NIR light-induced PDT and hypoxia-activated chemotherapy (Fig. 14b). The in vitro cytotoxicity of the system against 4T1 cells was then assessed using CCK-8 assay. Treatment with only NIR irradiation, UCD and UCS did not result in a significant decrease in the cell viability, implying the negligible toxicity of light irradiation and these NPs to 4T1 cells. Furthermore, the integration of the nanoplatform with anti-programmed death ligand 1 (α-PD-L1) therapy promotes a distant compartment effect that completely inhibits the growth of untreated distant tumors by generating specific tumor infiltration of cytotoxic T cells. Qu et al. have improved the tumor hypoxic microenvironment and enhanced the effect of PDT by increasing the oxygen in the tumor [125]. They modified Pt nano enzymes on the surface of PCN-224 to obtain a PCN-224-Pt composite, in which Pt nanoenzyme acts as a peroxidase-like enzyme can catalyze H_2_O_2_ to produce O_2_ with high hydrogen peroxide content, thereby improving the hypoxic environment of tumor tissues and enhancing the effect of PDT.

In addition to porphyrin-based NMOFs, Xie et al. synthesized BODIPY-immobilized Zr-based MOFs, namely UiO-PDT, by combining BODIPY ligands with UiO-66 via solvent-assisted ligand exchange [126]. Benefiting from the superior photosensitive properties of BODIPY ligand, the UiO-PDT nanocrystals have good biocompatibility and efficient ^1^O_2_ production, which can effectively kill cancer cells through PDT process. Zhou et al. used UiO-66 as a nanocarrier to modify PS and photocontrol molecular switches using coordination of Zr6 metal clusters in MOFs to achieve PDT, while controlling ^1^O_2_ production by adjusting the ratio of photocontrol molecular switches [127]. This study demonstrates the feasibility of in situ modification of multiple functional units in NMOFs nanoparticles. In addition, the stability of the prepared nano-agents was checked in aqueous media where PXRD and dynamic light scattering (DLS) data showed no significant disruption of the framework over a week. Tang et al. used NMOFs loaded with PSs to enhance the effect of PDT by simultaneously improving both the depth of laser penetration and the tumor hypoxic microenvironment. They used a one-pot method to synthesize ZIF MOFs loaded with both UCNPs and PSs methylene blue and further modified the nanoparticles with hydrogen peroxidase to obtain the core–shell nanocomposite [129]. They loaded both UCNPs and PSs into ZIF-8, and further modified the nanoparticles with peroxidase to obtain the core–shell nanocomposite UCNPs/MB@ZIF-8@catalase by one-pot method. Cell cytotoxicity assays of the nanocomposite and other contrast samples in dark were measured by MTT method against pancreatic cancer cell lines (PL 45 cells). All materials exhibit a relative low toxicity. The UCNPs can be used not only for NIR imaging, but also for ROS production under NIR light irradiation by FRET. Moreover, the modified peroxidase in UCNPs/MB@ZIF-8@catalase can catalyze endogenous H_2_O_2_ to produce oxygen in the tumor to overcome the two major obstacles of lack of oxygen in the tumor microenvironment and the limited depth of laser penetration during PDT.

### Photo-induced chemodynamic therapy (CDT)

**Fig. 15:**
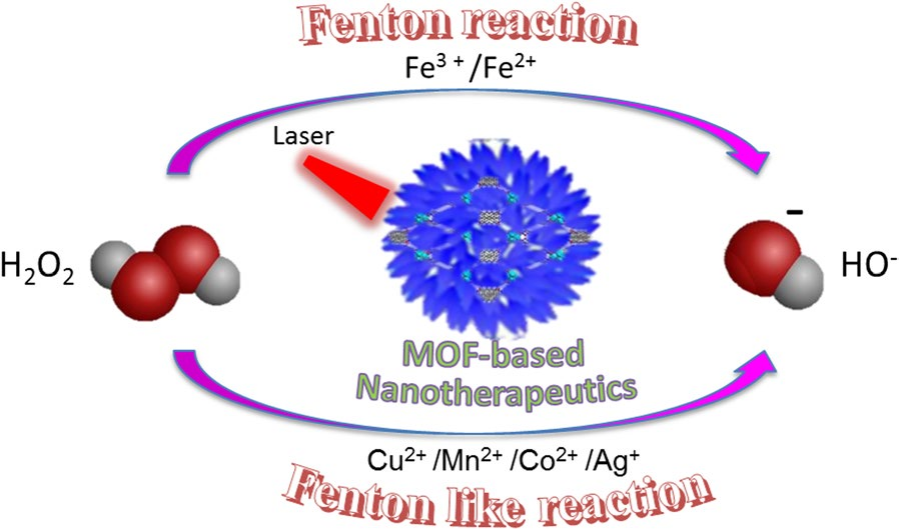
Mechanism of photo-driven Fenton or Fenton like reaction in photo-induced CDT by using MOF-based nanoplatform as therapeutic agent

CDT, a new tumor nano-catalytic therapy, not only has the advantage of high selectivity and low toxicity compared to PTT and PDT, but also overcomes the disadvantages of PDT which requires external energy for activation when treating deep-seated tumors [129–131]. The basic principle of CDT is to use nanotechnology to deliver Fenton or Fenton-like reaction catalysts into tumor tissues, catalyzing the over-expression of H_2_O_2_ in tumor cells to produce ·OH with strong oxidizing power, causing irreversible oxidative damage to molecules such as liposomes, DNA and proteins in tumor cells, thereby inducing apoptosis and killing tumor cells [132]. Therefore, many studies have focused on increasing CDT by improving the ability of tumor tissue to accelerate the Fenton reaction to produce ·OH. Most MOF structures with nodes of Fe and Cu have ligand bonds that are sensitive to pH and are prone to dissociation and release of metal ions in the slightly acidic environment of tumor tissue, while light-induced magnetic and photothermal heating can trigger a temperature rise within the tumor that generates sufficient cytotoxic ROS to amplify the CDT effect [133, 134]. Moreover, MOF materials can deliver large amounts of Fenton or Fenton-like reaction catalysts to tumor tissue, and the reaction rate can be further improved by photothermal effect. This offers the possibility of using MOF as catalysts for Fenton or Fenton-like reactions. So far, there are two ways to enhance CDT, one is raising the temperature at tumor tissue, another is enhancement of optical properties of materials (Fig. 15).

In 2019, Chen et al. synthesized an activatable nanoenzyme AMP by loading ABTS into the framework of MIL-100 using the Fe-based MOF material MIL-100 as a peroxidase-like enzyme and modifying it with PVP [113]. oxABTS and Fe^3+^ also depleted intracellular overexpressed GSH and reduced its consumption of MIL-100-catalyzed ·OH, thus enhancing CDT efficiency. In combination with PTT and CDT, AMP showed excellent in vitro and in vivo tumor suppressive effects. Compared to Fe-based catalysts, Cu-based Fenton-like reaction catalysts do not require excessively low pH conditions and have much higher catalytic activity than Fe-based catalysts, such as, Cu^+^ catalyzed H_2_O_2_ decomposition at 160 times the reaction rate of Fe^2+^ catalysts. Therefore, the use of Cu-based MOF materials to trigger CDT is expected to further improve therapeutic efficiency. Lin groups used Cu^2+^ and the tetracarboxyphenylporphyrin (H_4_TBP) to construct the nano-MOF material Cu-TBP for CDT, PDT and immuno-trimodal therapy of tumors [135]. In combination with the immunotherapeutic effect induced by the immune checkpoint inhibitor PDL-1, Cu-TBP not only eliminates tumors in situ but also inhibits the growth of distal tumors. This system provides inspiration for the design of MOF-triggered CDT-based combination therapy strategies.

**Fig. 16:**
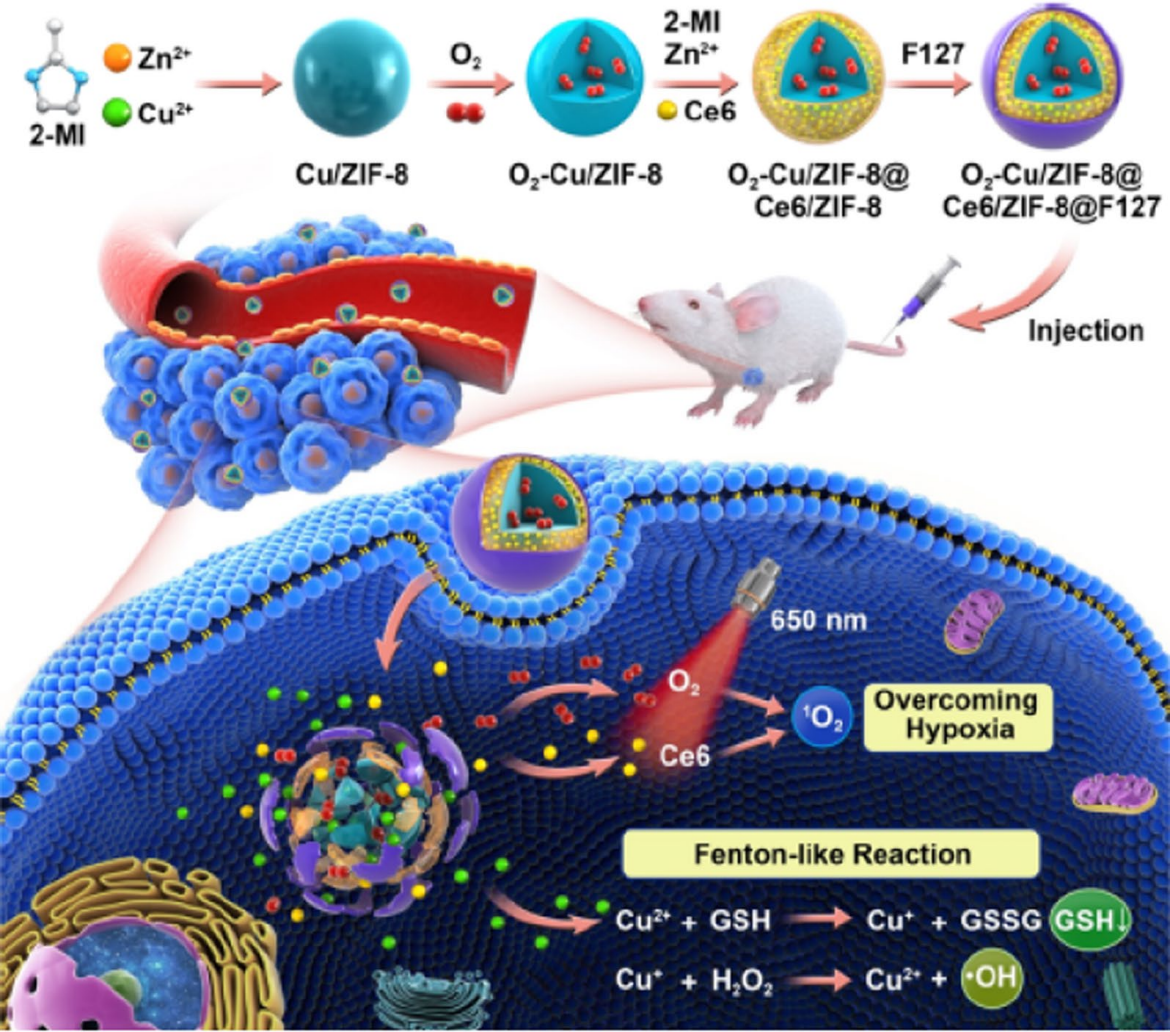
Schematic illustration of the fabrication process of the TME-responsive OCZCF nanoplatform for enhanced PDT and CDT through GSH depletion and O_2_ replenishment. Reproduced with permission [136]. Copyright 2020, American Chemical Society

High levels of glutathione (GSH) in tumor tissue deplete the ROS produced during CDT and PDT, thus reducing the therapeutic effect. Xie et al. constructed a combined ZIF-8-based CDT/PDT system with self-oxygenation, namely O_2_-Cu/ZIF-8@Ce6/ZIF-8@F127, which can release O_2_, Cu^2+^ and Ce6 when decomposed under microacidic tumour conditions (Fig. 16) [136]. The oxidation of Cu^2+^ consumes GSH in the tumor and is itself reduced to Cu^+^, which in turn catalyzes the decomposition of H_2_O_2_ in the tumour to produce ·OH. Moreover, under 650 nm laser irradiation, the released O_2_ could be converted to ^1^O_2_ by Ce6 to inhibited the growth of 4T1 transplanted tumors.

In summary, MOF materials can deliver large amounts of Fenton or Fenton-like reaction catalysts to tumor tissue, which can enhance the CDT effect by selective release of catalysts due to their acid response and other characteristics. However, there are still remains two bottlenecks in CDT: one is the efficiency of catalyzation in the acidic tumor microenvironment and another one is the low optimal reaction pH (2–4) as compared to tumor microenvironment. In addition, the efficiency of CDT is strongly influenced by the intra-tumoral H_2_O_2_ concentration, so increasing the concentration of H_2_O_2_ in tumor cells will help to improve the efficacy of CDT.

### Radiotherapy (RDT)

**Fig. 17:**
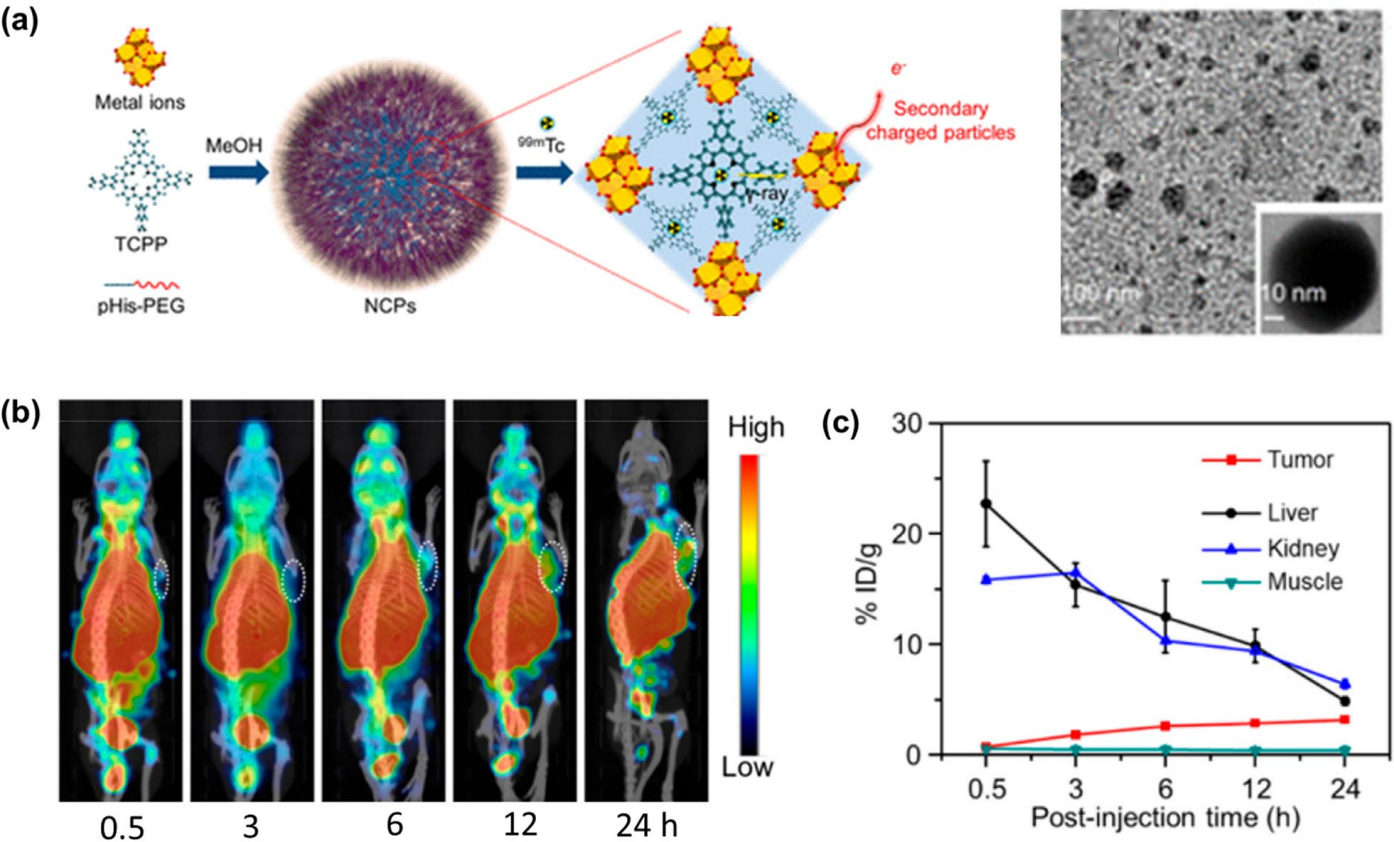
**a** Schematic illustration and TEM images of the synthesis of ^99m^Tc-Hf-TCPP-PEG. **b** in vivo SPECT images of 4T1 tumor-bearing mice after intravenous injection with ^99m^Tc-Hf-TCPP-PEG NCPs. **c** Quantification of SPECT signals in the liver, kidney, tumor, and muscle of 4T1 tumor bearing mice for mice at different time points after intravenous injection with ^99m^Tc-Hf-TCPP-PEG NCPs. Reproduced with permission [139]. Copyright 2018, American Chemical Society

RDT is a treatment process that uses scintillators and heavy metal materials to absorb X-ray energy and transfer the energy to a photosensitizer to stimulate the production of oxygen radicals by the photosensitizer for cancer treatment [137, 138]. Metal atoms with high atomic number have been shown to possess high X-ray absorption coefficients. Liu et al. prepared polyethylene glycol (PEG) modified MOF-based nanoplatform, namely Hf-TCPP-PEG, composed of Hf^4+^ and TCPP by a one-pot method, in which Hf^4+^ with high atomic number served as a radiotherapy sensitizer to improve the efficiency of radiotherapy (Fig. 17a) [139]. In addition, by chelating TCPP with ^99m^Tc^4+^, a ^99m^Tc^4+^ labelled RDT agent ^99m^Tc-Hf-TCPP-PEG was obtained, which could generate charged particles for radiosensitisation and kill cancer cells by X-rays emitted by Hf^4+^ and ^99m^Tc (Fig. 17b and c). Subsequently, Lin et al. found that ROS production was inversely proportional to particle size [140]. They concluded that a larger specific surface area could be an important design and accordingly developed Hf-based NMOFs (Hf_6_-DBA and Hf_12_-DBA), both of which have high Z-element and high specific surface area, making them ideal radiation-enhanced materials, while the unique porous structure of the Hf-based NMOFs could facilitate the diffusion of the generated short-lived ROS for better cytotoxic effects. In vitro and in vivo experiments showed that both Hf_6_-DBA and Hf_12_-DBA were better than HfO_2_ NPs in terms of radiation enhancement at the same dose of Hf. Furthermore, the radioluminescence data of the two MOF materials indicate that Hf_12_-DBA is a better radioactive enhancer than Hf_6_-DBA, which may be due to the fact that the Hf_12_ cluster absorbs X-rays more efficiently than the Hf_6_ cluster.

**Fig. 18:**
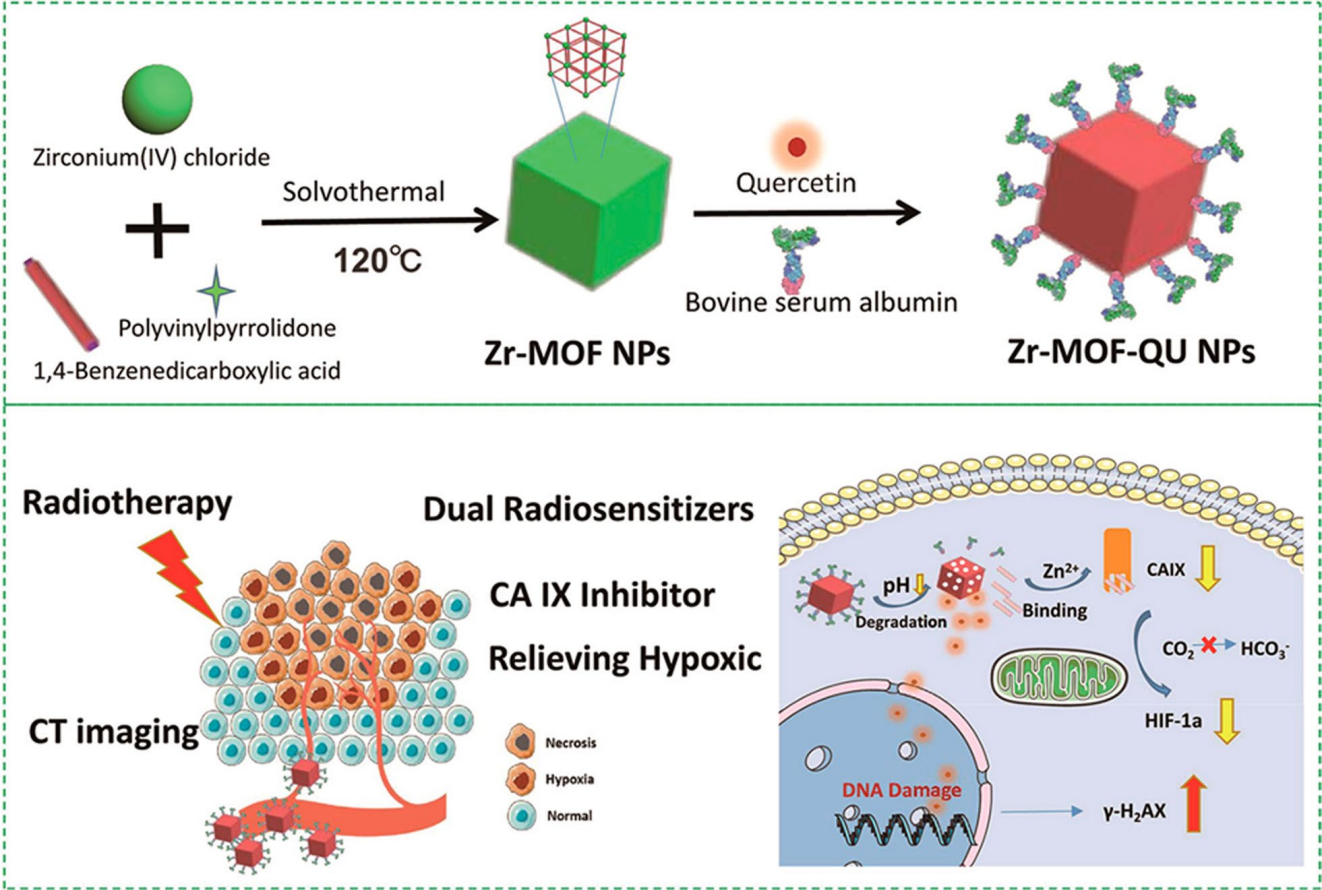
Schematic illustration of the fabrication process of the Zr-MOF-QU nanoplatform for enhanced RDT and CDT through 1,4-benzenedicarboxylic acid depletion and relieving hypoxia in the tumor microenvironment. Reproduced with permission [142]. Copyright 2019, American Chemical Society

Tumor hypoxia plays a key role in radiation resistance, and one strategy to improve the efficacy of RDT is to modulate the radiosensitivity of the tumor microenvironment [141]. By using ZrCl_4_ and 1,4-benzenedicarboxylic acid as precursors, Meng et al. prepared Zr-MOF by a solvothermal method, followed by QU loading and BSA modification to obtain Zr-MOF-QU nanomaterial for RDT (Fig. 18) [142]. Stability experiments showed that Zr-MOF-QU was stable under normal physiological conditions and was dissociated into Zr^2+^ and 1,4-benzenedicarboxylic acid in an acid tumor microenvironment, so that when exposed Zr-MOF-QU to the tumor site, 1,4-benzenedicarboxylic acid is broken down from Zr-MOF and bound to Zn^2+^ of CAIX to inhibit its catalytic activity. Zr-MOF-QU provides synergistic effects to radiosensitize and modify the radiation resistance of cancer cells, enabling a dual-sensitized tumor radiation therapy. In addition to O_2_, NO can also help cells cope with hypoxia. Recently, Hu et al. loaded d-arginine (d-Arg) into metal–organic backbone MIL-100 (Fe) nanoparticles, where Fe^3+^ ions can react with H_2_O_2_ through the Fenton reaction, generating free radicals that may act synergistically with d-Arg-derived NO to reduce hypoxia and kill tumors [143]. The results showed that the d-Arg-loaded nanoparticles not only enhanced tumor killing but also effectively avoided tumor metastasis to the lung after radiation treatment.

Brief in all, although NMOFs could act as a promising radiation enhancer due to their superior controllability in terms of metal centers, ligands, size and porous structure. However, the investigation of the relationship between the tumor microenvironment and the efficiency of radiotherapy, and the integration of radiotherapy with other therapeutic approaches are all key to further improving the efficacy of cancer treatment.

## Conclusion and outlook

Currently, light-mediated therapy is an emerging method of tumor treatment, which is characterized by non-invasive, highly selective and low toxic side effects by transporting a photosensitizer or photothermal reagent to the tumor site and using an external laser to precisely irradiate the tumor site for tumor suppression. The therapeutic effect of MOFs on cancer cells and tumors is due to its highly porous biocompatible customizable hybrid structure with the ability to load drugs, proteins, genes, etc. Moreover, the easy functionalization of NMOFs to improve the anti-tumor effects of optical therapeutic platforms by improving laser penetration depth, targeting effect, imaging guidance or combination therapy, providing new ideas and strategies for the treatment of tumors. At present, research on NMOFs is focused on the design, development and commercialization of NMOFs rather than the design and synthesis of crystal structures. Fabrication of composites is a good way to explore other ranges of NMOFs, particularly for imaging and drug delivery applications in the diagnosis and treatment of cancer. Stability, biocompatibility, toxicity and targeted release of drugs are some of the key challenges in developing good MOFs for biological applications.

In terms of diagnosis, despite the fact that some Gd^3+^, Mn^2+^, and Fe^3+^-containing MOFs have shown good efficacy for MRI; the high-Z with strong X-ray attenuation properties (e.g., Hf and Zr) have also act as viable CT contrast agents, but more efforts are required to improve the efficiency of imaging, such as minimizing the signal-to-background ratio, which is a very important medical imaging index. In addition, the complexity of the body or cellular environment brings challenges for the delivery and precise release of drugs at the desired site of action. The transport of drug system across biological membranes, including blood brain barrier, is also a major challenge, and much effort is needed in this area to improve the prospects of NMOFs and MOFs in the diagnosis and treatment of cancer.

In terms of therapy, research on light-mediated therapeutic research based on NMOFs up a new avenue to address current technical barriers, but enables safer treatment for patients, and further exploration is needed to rationalize the clinical application of these therapeutic nano-agents in the tumor microenvironment. The toxicological behavior of MOF-based agents in the human vascular system in terms of potential immune response, biodistribution and toxicity in organs, and excretion in the liver, bile and kidneys need to be further explored. Furthermore, a systematic understanding of the human body’s response to different external stimuli (e.g., light, ultrasound, etc.) is essential, which could provide important insights for the fabrication of appropriate photoactivation platforms for therapeutic use in living systems.

Overall, despite many efforts to improve the performance of current MOF-based nano-therapeutic agents, it is still not ready to enter a new phase of clinical trials. The development of new MOF-based nano-agents requires full consideration of specificity, selectivity, efficiency, degradability, responsiveness, delivery, targeting and biosafety. Meeting all these requirements regarding the development of the next generation of MOF-based nano-therapeutic agents will certainly accelerate the pace of clinical translation significantly.

## Data Availability

Not applicable.

## Abbreviations

NMOFs: Nanoscale metal–organic frameworks
EPR: Enhanced permeability and retention
PS: Photosensitizer
*S*_0_: PS ground state
*S*_1_: PS excited singlet state
*T*_1_: PS-excited triplet state
ROS: Reactive oxygen species
MRI: Magnetic resonance imaging
CT: X-ray computed tomography
PET: Positron emission tomography
PAI: Photoacoustic imaging
*T*_1_: Longitudinal relaxation rates
*T*_2_: Transverse relaxation rates
FL: Fluorescence
PAT: Photothermal ablation therapy
LD50: Lethal dose 50
SPIOs: Superparamagnetic iron oxides
DTPA: Diethylenetriaminepentaacetic acid
TCPP: Tetrakis (4-carboxyphenyl) porphyrin
PB: Prussian blue
BODIPY: Iodine-boron-dipyrromethene dye
SBUs: Secondary building units
NIR: Near infrared
RhB: Rhodamine B
MGC-803: Mouse gastric cancer 803
HASMC: Human airway smooth muscle cells
PersL: Persistent luminescence
MTT: 3-(4,5-Dimethylthiazol-2-yl)-2,5-diphenyltetrazolium bromide
TCH: Tetracycline hydrochloride
5-FU: 5-Fluorouracil
PDT: Photodynamic therapy
DCA: Dichloroacetate
TPP: Triphenylphosphonium
PEG: Polyethylene glycol
FA: Folic acid
PDA: Polydopamine
THQ: Tetrahydroxyanthraquinone
PVP: Polyvinylpyrrolidone
UCL: Upconversion luminescence
ICG: Indocyanine green
UCNPs: Upconversion nanoparticles
PTT: Photothermal therapy
CDT: Chemodynamic therapy
PDIs: Perylenediimide
PTAs: Photothermal agents
HA: Hyaluronic acid
ABTS: 2,2′-Azino-bis(3-ethylbenzothiazoline-6-sulfonic acid
LSPR: Localized surface plasmon resonance
H_2_DBP: 5,15-Di(p-benzoato)porphyrin
H_2_DBC: 5,15-Di(p-benzoato)-chlorin
TPZ: Tirapazamine
α-PD-L1: Anti-programmed death ligand 1
H_4_TBP: Tetracarboxyphenylporphyrin
GSH: Glutathione
